# Electrolyte‐Dependent Sodium Plating for Anode‐Free Na‐Ion Batteries Studied by Operando Optical Microscopy

**DOI:** 10.1002/advs.202600058

**Published:** 2026-02-20

**Authors:** Moritz Exner, Dominik Stepien, Annica I. Freytag, Pedro B. Groszewic, Xiangping Min, Nour Adrah, Peter Axmann, Philipp Adelhelm

**Affiliations:** ^1^ Institut für Chemie Humboldt‐Universität zu Berlin Berlin Germany; ^2^ Joint Research Group Operando Battery Analysis (CE‐GOBA) Helmholtz‐Zentrum Berlin für Materialien und Energie Berlin Germany; ^3^ Department Spins in Energy Conversion and Quantum Information Science (SE‐ASPIN) Helmholtz‐Zentrum Berlin für Materialien und Energie Berlin Germany; ^4^ Department of Radiation Science and Technology Delft University of Technology Delft The Netherlands; ^5^ ZSW Center for Solar Energy and Hydrogen Research Baden‐Württemberg Ulm Germany; ^6^ Center for the Science of Materials Berlin (CSMB) Humboldt‐Universität zu Berlin Berlin Germany

**Keywords:** anode‐free, dendrites, electrolyte, Na‐ion batteries, sodium

## Abstract

Anode‐free sodium ion batteries (SIBs) promise higher energy density and lower costs, by eliminating the need for an anode host material; however, achieving efficient Na plating/stripping remains a major challenge. Here, three electrolyte classes − carbonate‐based, glyme‐based, and a localized high‐concentration electrolyte−are evaluated for Na plating/stripping on a commercial carbon‐coated aluminium current collector. Measurements across a broad temperature and current range (−30°C–+60°C, 0.25–14 mA cm^−2−^) and studies on the Na growth modes by operando optical microscopy reveal the superior behavior of the glyme‐based electrolyte, including a uniform crystalline metal deposition. In anode‐free full cells with Na_4_Fe_3_(PO_4_)_2_P_2_O_7_ as cathode, this electrolyte enables superior cycling with 75.3% capacity retention over 400 cycles at areal loadings above 3 mAh cm^−2−^. Projected energy densities of 290 Wh/kg and 751 Wh/l are calculated at the cell‐stack level, exceeding current LiFePO_4_‐based Li‐ion batteries. The excellent Na plating/stripping behavior is evidenced by a particularly low initial areal capacity loss (IACL, mAh cm^−2^). The IACL parameter represents the first cycle Na inventory loss that must be compensated by the cathode. Unlike for conventional Na‐ion cells with traditional anodes, the IACL is a constant for anode‐free cells. For the given cell, the IACL amounts to only 0.14–0.16 mAh cm^−2^ (~5% of the 3 mAh cm^−2^ cathode areal capacity). This highlights the potential of anode‐free SIBs using commercially available components.

## Graphical abstract

Anode‐free sodium‐ion batteries are examined using carbonate‐, glyme‐, and localized high‐concentration electrolytes. Distinct sodium plating morphologies are identified by operando optical microscopy, revealing uniform crystalline deposition for glyme‐based systems. This enables stable cycling of high‐areal‐loading NFPP full cells with low initial sodium loss and high projected cell‐stack energy densities, highlighting the promise of commercially compatible anode‐free battery designs.

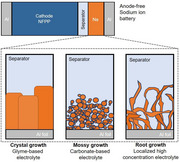

## 1 Introduction

While lithium‐ion batteries (LIBs) presently dominate the market for rechargeable batteries, efforts to improve their energy density and to identify more cost‐effective storage solutions continue to be major research focuses. For example, the dependence on lithium and critical transition metals such as Ni or Co, together with manufacturing constraints, emphasizes the need for sustainable and alternative battery technologies [1, 2]. Sodium is far more abundant than lithium, making sodium‐ion batteries (SIBs) attractive for cost‐effective energy storage [3]. While a wide range of cathode active materials (CAMs) has been explored for Na‐ion batteries, including various layered oxides, polyanionic compounds, and Prussian blue analogs (PBAs), the selection of suitable anode active materials (AAMs) remains comparatively limited [4]. Graphite and silicon, which are widely used in LIBs, are largely inactive for Na storage. Graphite may be used when using electrolytes containing co‐intercalating solvents; the capacity is, however, limited to about 110 mAh g^−^^1^ so far [5].

An alternative approach involves alloy‐type anode materials such as tin (Sn), antimony (Sb), bismuth (Bi), and their compounds. They provide high theoretical capacities of several hundred mAh g^−^^1^ (847 mAh g^−1^ for Na_15_Sn_4_) but suffer from severe volume expansions up to 400% and hence rapid capacity fading during cycling [6]. As a result, hard carbons have emerged as the most commonly used anode materials for Na‐ion batteries, functioning as an insertion‐type material similar to that in Li‐ion batteries. Commercial hard carbons typically deliver capacities of around 300 mAh g^−^^1^ and can be produced from renewable precursors, though their volumetric capacity remains relatively limited [3, 7, 8, 9, 10, 11]. Overall, the limitations of anode active materials in SIBs are a key factor behind their lower energy density compared to LIBs.

Maximizing the energy density of SIBs could be achieved by employing sodium metal as the anode (1166 mAh g_Na_^−^^1^), which could enable energy densities comparable to or even higher than those of current LIBs [7, 12], see also the results section.

The most desirable use of Na metal as an anode is the “anode‐free” configuration. In this design, cells are assembled containing only the CAM (and no AAM). The Na‐anode forms during cell charging by plating Na directly onto the current collector. The concept is also referred to as “zero excess”‐design to distinguish it from Na‐metal batteries (SMBs), which typically are assembled with an oversized “excess” Na foil as AAM [13]. The anode‐free design also has the advantage of simplifying manufacturing and allows loading a higher fraction of CAM into the cell [3, 14]. As the sodium inventory is entirely provided by the CAM, achieving highly efficient and reversible sodium plating/stripping is crucial for the practical realization of this concept.

Like for lithium, the use of sodium metal anodes faces critical challenges such as uneven plating, dendrite growth, and the accumulation of “dead sodium” that is electronically isolated from the current collector [15]. To mitigate these issues, several strategies have been explored, including the use of composite anodes, coatings, 3D structures, or the use of optimized electrolyte formulations [16]. Although interfacial modifications and coatings may enhance stability, they also add complexity and manufacturing costs, which calls for more efficient approaches to stabilize Na plating. Additionally, Cu foil has been commonly used as the current collector in anode‐free cells [17, 18]. Unlike Li, Na does not form alloys with Al at low potentials, therefore, Al can also be used as the anode‐side current collector. Particularly in light of rising copper prices, the use of Al is highly attractive and cost‐effective, as Al foil costs only one‐quarter of a comparable Cu foil [19].

Electrolyte optimization is a promising strategy to enable uniform Na plating and stable cycling, which may be particularly crucial for advancing anode‐free SIBs [3]. Carbonate‐based electrolytes dominate commercial LIBs owing to their wide electrochemical stability window, high ionic conductivity, and low viscosity. Favorable properties are obtained by mixing different types, such as dimethyl carbonate (DMC), propylene carbonate (PC), ethylene carbonate (EC), diethyl carbonate (DEC), or ethyl methyl carbonate (EMC) [20]. They are also widely used in electrolytes for SIBs, for which even higher ionic conductivities can be found compared to LIBs, thanks to the lower charge density of Na^+^ compared to Li^+^. For example, 12 mS cm^−1^ are found for 1 m NaClO_4_ dissolved in EC:PC:DMC (0.45:0.45:0.1 wt.%) while at the same time providing a wide electrochemical stability window [21, 22, 23]. Unfortunately, the use of carbonates in anode‐free SMBs leads to very poor performance. For example, Seh et al. reported a Coulomb efficiency (CE) below 25% with EC/DEC and EC/DMC mixtures [17].

Glycol dimethoxy ethers (also called glymes), such as monoglyme (1G) and diglyme (2G), have recently attracted attention across various battery chemistries, including SIBs [24]. Glymes exhibit exceptional stability against reduction, and their ether groups are able to chelate with metal cations, forming crown ether‐like complexes. This enables good salt solubility despite showing comparatively low relative permittivity. With diglyme, the most common solvation conformation is Na^+^(diglyme)_2_ [25]. Highly reversible Na plating and stripping have been demonstrated with glyme‐based electrolytes. Seh et al. showed that a Cu/Na half‐cell with 1 m NaPF_6_ in 2G achieves 99.9% CE over 300 cycles, enabled by the formation of a uniform and compact SEI [17, 26]. Therefore, unlike Li metal, fully compatible electrolytes remain elusive for Na despite the higher reactivity. Zhou et al. later attributed this stability to the ability of diglyme to bond strongly with the Na‐ion, while the PF_6_^−^ anion can stay far from the ion and the electrode surface, thereby suppressing corrosion [27]. Consistently, Ma et al. observed shiny, smooth deposits by optical microscopy, further confirming the beneficial plating behavior in glyme‐based electrolytes [18, 26]. NaPF_6_ is the most common sodium salt with carbonate‐ and ether‐based solvents due to its high ionic conductivity and good compatibility, but its solubility in ether‐based electrolytes (around 2 m) limits its use in HCEs [20].

Electrolyte concentrations play a decisive role in Na plating behavior. High concentration electrolytes (HCEs, > 3 m) have recently shown their ability to suppress dendrite growth and maintain a stable high‐voltage performance [28]. In HCEs, most solvent molecules are coordinated to cations, while anions form contact ion pairs (CIPs) and aggregates (AGGs). This leads to an anion‐enriched solvation environment, which facilitates the formation of robust, inorganic‐rich SEI/CEI layers, effectively passivating electrode interfaces [20]. However, their practical use is limited by high viscosity, poor wettability, and the high cost of salts. To address these issues, localized high‐concentration electrolytes (LHCE) employ an inert diluent to reduce viscosity and improve wettability while preserving the beneficial solvation structure of HCEs [29]. The inert solvent does not disrupt the local solvation structure and retains the beneficial CIP/AGG‐rich coordination environment. The solvation shells are spatially heterogeneous with locally concentrated Na solvation and a dilute dielectric background. This structure promotes preferential anion reduction at the interfaces and forms a robust inorganic SEI/CEI layer.

Typical LHCE for batteries with Na metal as anode rely on NaFSI as the salt due to its high solubility, while NaPF_6_ cannot reach concentrations of 3 m in glyme‐based solvents. 1G is used as the solvent due to its low viscosity, and bis(2,2,2‐trifluoroethyl) ether, and 1,1,2,2‐Tetrafluoroethyl 2,2,3,3‐tetrafluoropropylether (TTE) are used as inert diluents [20, 30]. Wang et al. demonstrated a NaFSI/1G/TTE (1:1.5:1.5 molar ratio) LHCE that enabled stable cycling in symmetric Na cells, albeit at a low current density and plating capacity (0.2 mA cm^−2^, 0.2 mAh cm^−2^), and achieved an average CE of 98.4% for Na vs NVP full cells [29]. Notably, truly anode‐free practical demonstrations remain scarce in the literature, as most reported studies rely on specialized or precycled anode‐less configurations or employ low areal loadings [18, 31].

In this work, we investigate the influence of the electrolyte on the microscopic morphology evolution of the plated/stripped sodium, which can be directly monitored using operando optical microscopy. Three representative electrolytes belonging to the carbonate, ether, and localized high concentration electrolyte (LHCE) classes are systematically compared in otherwise identical cell configurations, see Table 1. The differences between these electrolytes are highlighted by using various techniques, including Raman spectroscopy, viscosimetry, electrochemical impedance spectroscopy (EIS), differential scanning calorimetry (DSC), cyclic voltammetry (CV), and pulsed‐field gradient nuclear magnetic resonance (PFG‐NMR). Furthermore, low‐temperature properties were evaluated down to −30°C, demonstrating a crucial advantage over conventional Li‐ion systems. The electrolytes are then evaluated using two‐ and three‐electrode cells using commercially available, carbon‐coated aluminum foil (C‐Al) as a current collector to assess the cycling stability. The morphology of the Na deposition is studied by operando optical microscopy and by scanning electron microscopy (SEM). Side reactions and impedance growth are further analyzed using impedance spectroscopy. Finally, to evolve from the micro stage of dendrite morphology to the macro stage of complete working batteries with high capacities, the electrolytes are tested in anode‐free full cells with commercial Na_4_Fe_3_(PO_4_)_2_P_2_O_7_ (NFPP) as CAM, demonstrating a capacity retention of 71.6% after 400 cycles with a diglyme‐based electrolyte, with additional tests confirming stable operation at areal loadings above 3 mAh cm^−2^. An energy comparison shows that such anode‐free cells with NFPP as CAM may provide energy densities significantly above current SIB technology.

**TABLE 1:** Overview of the three electrolytes studied: glyme‐based, carbonate‐based, and localized high concentration electrolyte (LHCE) along with their composition regarding salts, solvents, and abbreviations.

Abbreviation	Composition	Salt	Solvent	
NaPF‐2G	1 m	Sodium hexafluorophosphate (NaPF_6_)	Diglyme (2G)	
NaPF‐EC/PC	1 M in 1:1 (wt.%)	Sodium hexafluorophosphate (NaPF_6_)	Ethylene carbonate (EC)	Propylene carbonate (PC)
LHCE	1:1.5:1.5 (mol.)	Sodium bis(fluorosulfonyl)imide (NaFSI)	Dimethoxyethane (1G)	1,1,2,2‐Tetrafluoroethyl 2,2,3,3‐tetrafluoropropylether (TTE)

## 2 Results and Discussion

### 2.1 Physicochemical and Electrochemical Characterization of the Electrolytes

Fast ion diffusion, low viscosity, and high conductivity of the electrolytes are important to provide cell function at high current densities and low temperatures, while a good reductive stability is important to limit electrolyte decomposition and SEI formation on the anode. In this section, we explore these key parameters for the three electrolytes.

To analyze the solvation of the three electrolytes, see Table 1, Raman spectra were recorded. In the presence of NaPF_6_, a shift of the C─O─C stretching and CH_2_ rocking modes of diglyme in the region between 800 and 900 cm^−1^ toward higher wavenumbers is observed, indicating partial coordination of the ether groups to Na^+^ (Figure S1) [28, 32, 33]. For the carbonate‐based electrolyte, besides the peak at 894.5 cm^−1^ of pure EC, a new peak develops upon solvation at 901.3 cm^−1^, attributed to the ring enlargement stretching vibration of the EC‐Na^+^ solvate [28, 34]. Similarly, PC and EC exhibit skeletal bending modes at 714 and at 717 cm^−1^, which shift to higher wavenumbers upon Na^+^ coordination [35, 36]. In contrast, TTE acts as an inert diluent in the LHCE system, as it does not alter the position or shape of the characteristic peaks of monoglyme or the NaFSI salt [37].

The total symmetric stretching mode of PF_6_^−^ in the pure salt appears at 765 cm^−1^ and shifts to lower frequencies [38]. For NaPF‐2G, it is shifted to 741 nm. With NaPF‐EC/PC, the signal is at 742.5 cm^−1^. In the LHCE, the signal of the FSI^−^ anion is shifted from 755 to 746 cm^−1^. Such peak shifts provide insight into the solvation environment: in systems with the same salt and concentration, higher wavenumbers generally indicate stronger ion–solvent–cation interactions. Accordingly, the carbonate‐based electrolyte shows slightly stronger interactions than the glyme‐based system. In LHCEs, however, the interpretation is more complex since the high salt concentration also promotes extensive ion pairing and aggregation, in addition to solvent effects. The pronounced shift observed for LHCEs, therefore, reflects both the strong solvation structure and the concentration‐induced coordination environment [28].

**FIGURE 1:**
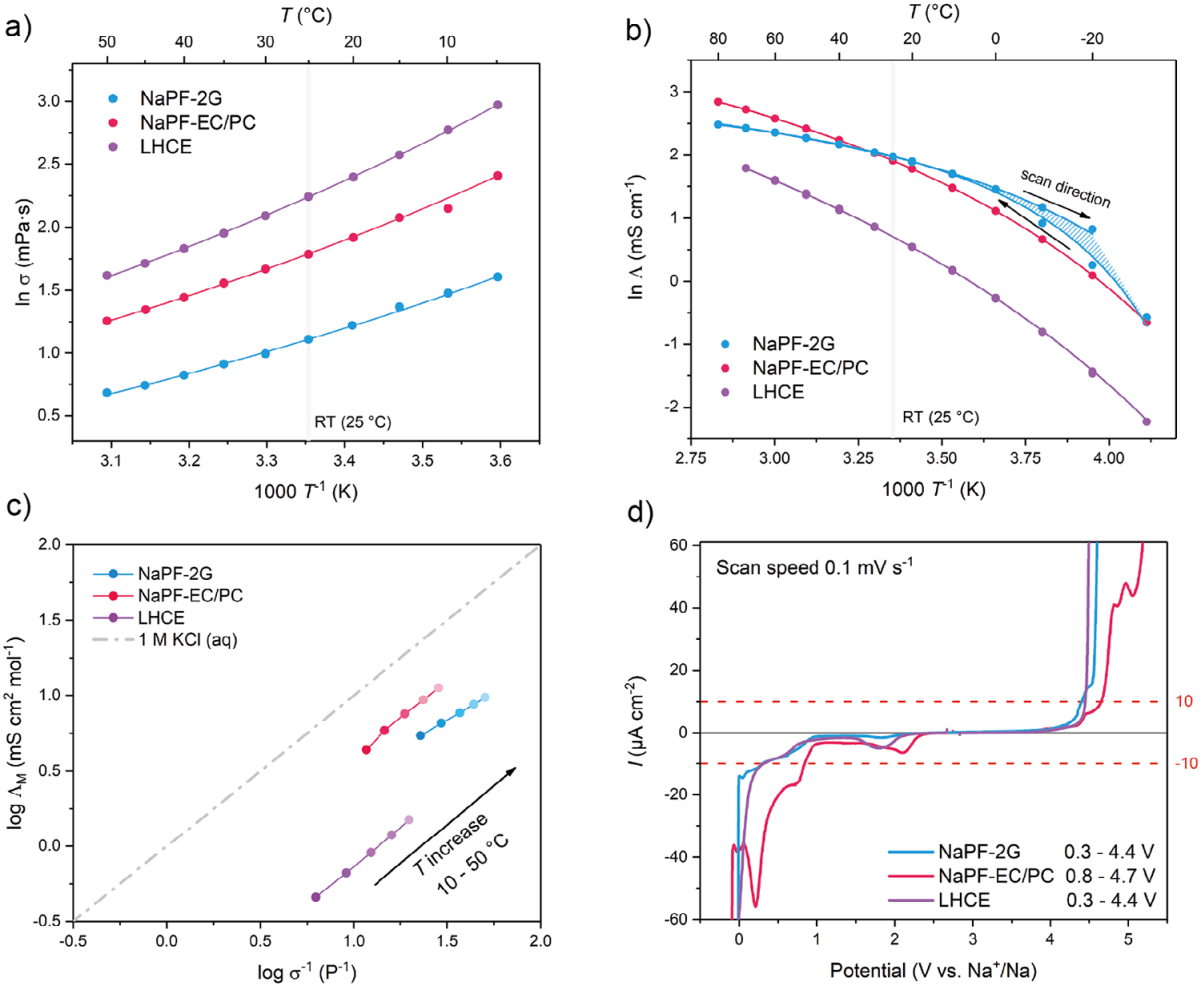
Physicochemical properties of the NaPF‐2G (blue), NaPF‐EC/PC (red), LHCE (violet) electrolytes: a) Viscosity in the range of 5°C–50°C, b) ionic conductivity in the range of −30°C–80°C, c) Walden plot for temperatures between 10°C and 50°C. The dashed line (gray) represents the ideal 1 m aqueous KCl reference. d) LSV at room temperature in three‐electrode‐cells with a scan speed of 0.1 mV s^−1^ and cut‐off currents of 10 µA cm^−2^ used to determine the ESW.

Electrolyte viscosity was determined in the range of 5 – 50°C, and the Arrhenius plots are shown in Figure 1a. At 25°C, the dynamic viscosities are 3.0, 7.0, and 9.4 mPa·s for 2G, EC/PC, and LHCE, respectively, with all electrolytes showing decreasing viscosity at higher temperatures.

The data exhibit curvature and were fitted using the Vogel–Tamman–Fulcher (VFT) equation:

(1) $$\eta =\eta_{0}\;e^{\frac{B}{T-T_{0}}}$$

where B is the pseudo‐activation energy, T is the current temperature, and *T*_0_ is the pseudo‐ideal glass transition temperature. As described by Morales et al., the logarithmic form of the VFT was used to better fit data spanning several orders of magnitude [21]. Fitting parameters are provided in Table S1. LHCE has the highest pseudo activation energy of 4.0 kJ mol^−1^, compared to 3.4 kJ mol^−1^ with NaPF‐EC/PC, and 3.0 kJ mol^−1^ with NaPF‐2G, indicating that its viscosity is most sensitive to temperature changes [21].

The Arrhenius plots for the electrolytes are shown in Figure 1b. Conductivity depends on the solvent's dielectric constant, salt dissociation, solution viscosity, and salt concentration. The curved temperature dependence observed in Figure 1b was fitted using the VFT equation for conductivity:

(2) $$\sigma =\sigma_{0}\;e^{\frac{-B}{T-T_{0}}}$$

The fitting parameters are listed in Table S2. At 25°C, the conductivities are 7.10, 6.75, and 2.03 mS cm^−1^ for NaPF‐2G, NaPF‐EC/PC, and LHCE, respectively, and align well with literature values [21, 22, 37]. While 2G and EC/PC have relatively similar conductivity, the latter is more strongly affected by temperature, reflecting the greater influence of viscosity, consistent with higher viscosity measured for EC/PC. LHCE shows lower conductivity, although it has the highest charge carrier concentration. This is attributed to its higher viscosity and the prevalence of ion‐pair aggregates, which reduces the number of mobile ionic species. Additionally, the inert TTE diluent likely interrupts the cation‐hopping, commonly observed in ionic liquids and highly concentrated electrolytes, further limiting conductivity [37, 39].

The VFT‐derived activation energies range from 1.2 to 5.0 kJ mol^−1^, indicating significant salt dissociation in all three electrolytes, different from, for example, super‐concentrated electrolytes where full dissociation is not possible [40]. NaPF‐2G shows hysteresis at low temperatures between cooling and heating, suggesting a possible phase transition. With [(diglyme)_2_Li^+^]PF_6_ complexes, a phase transition at 22°C was observed, which reflects the onset of structural disorder in the 3D arrangement of cation–anion network [41]. Such hysteresis typically occurs near phase transition, crystallization, or melting points, with shifts to lower temperatures during cooling and towards higher temperatures during warming [42]. Differential scanning calorimetry (DSC) conducted at scan rates of 1–10°C min^−1^ (Figure S2) revealed no phase transitions between −40°C and 30°C for any electrolytes. The DSC measurements also proved that PC as a co‐solvent can efficiently lower the solidification point below −40°C compared to the solidification point of pure EC at 36.4°C [22].

The relationship between viscosity and conductivity can be shown by following the Walden rule:

(3) $$\log\left(\Lambda \right)=\log\left(\frac{1}{\mu }\right)\;$$

The Walden plot for the studied electrolytes is shown in Figure 1c. Ionicity is determined from the vertical distance (C value) between measured points and the KCL line, yielding 21% for NaPF‐2G, 39% for NaPF‐EC/PC, and 7% for LHCE [43]. All points lie below the KCl line, indicating non‐ideal dissociation, consistent with dynamic hindering effects such as ion pairing that reduce net charge transport [21]. Similar results for EC/PC were obtained by Morales et al. [21] and Chayambuka et al. [44]. Despite its higher viscosity, EC/PC exhibits the highest ionicity and lies closest to the KCl line, reflecting better salt dissociation, which can be attributed to the high dielectric constant of EC (89.78) and PC (64.92) compared to 2G (7.23) [22, 45]. LHCE shows the lowest ionicity, consistent with the high prevalence of ion aggregates in the system.

The electrochemical stability of the electrolytes was evaluated using linear sweep voltammetry with C‐Al as working electrode and Na as the counter and reference electrode, and a cutoff current of 0.01 mA cm^−^^2^, corresponding to the onset of electrolyte decomposition [46]. The resulting electrochemical stability windows (ESW) are shown in Figure 1d.

NaPF‐2G and LHCE show similar ESW, ranging from 0.3 to 4.4 V, while NaPF‐EC/PC has a higher oxidative stability, up to 4.7 V vs Na^+^/Na. However, EC/PC has a limited reduction stability, only down to 0.8 V, which poses a challenge for Na metal batteries as the Na deposition happens at around 0.0 V Na^+^/Na [22, 47]. These results suggest that 2G and LHCE are suitable for Na metal batteries with cathodes operating below 4.4 V, while the EC/PC is likely to undergo significant degradation at the Na‐plated anode. All electrolytes display small currents below 2.5 V during the initial reductive scan, attributable to electrolyte decomposition (SEI formation as the dominant factor) and Na insertion into the carbon coating of the aluminium current collector, with reversible electrodeposition beginning near 0 V [48, 49]

The self‐diffusion coefficients of solvent, cation, and anion were determined using pulsed field gradient nuclear magnetic resonance spectroscopy (PFG‐NMR), see Table 2. The measured diffusion coefficient (*D* values) of the solvents represents an average of both free and coordinated molecules within the electrolytes [21]. Among all nuclei, the diglyme of the NaPF‐2G electrolyte had the highest self‐diffusion values, while the LHCE showed the lowest. Remarkably, the ^1^H diffusion coefficient of 2G is five times higher than that of 1G, although earlier studies reported higher diffusion in 1G when compared at similar salt concentrations [50]. Since diffusion coefficients are related to viscosity, ionic radius, and temperature via the Stokes–Einstein relation, the enhanced mobility observed here is most likely a consequence of the lower viscosity of the 2G electrolyte [39]. EC has a larger *D* value (2.68 10^−10^ m^2^ s^−1^) than PC (2.20 10^−10^ m^2^ s^−1^), which, despite its smaller molecular size, may indicate a weaker Na‐ion‐EC interaction, compared to PC [21]. In both electrolytes, the PF_6_ anion diffuses faster than Na^+^. The sodium ion has a greater charge density compared to the counter ion. This leads to greater solvation and slower dynamics for the Na cation. This trend was also observed by Morales et al. [21]. In the LHCE, TTE shows significantly faster diffusion than either ion or 1G, which is consistent with Raman results, and indicates that TTE does not participate in the primary Na‐ion solvation shell [39]. For ^23^Na, the large quadrupole moment combined with the high viscosity of the LHCE electrolyte led to a poor signal‐to‐noise ratio [50].

**TABLE 2:** Room‐temperature ^1^H, ^19^F, and ^23^Na self‐diffusion parameters *D* of NaPF‐2G, NaPF‐EC/PC, and LHCE calculated by PFG‐NMR. *D* (^23^Na) could not be obtained for LHCE.

	*D* (^1^H) (10^−10^ m^2^ s^−1^)		*D* (^19^F) (10^−10^ m^2^ s^−1^)		*D* (^23^Na) (10^−10^ m^2^ s^−1^)
NaPF‐2G	4.35 (2G)		2.8 (PF_6_^−^)		2.2
NaPF‐EC/PC	2.68 (EC)	2.2 (PC)	1.94 (PF_6_^−^)		1.3
LHCE	0.78 (1G)	1.82 (TTE)	0.67 (FSI^−^)	1.94 (TTE)	—

Given the fact that NaPF‐2G offers fast ion diffusion with good conductivity and low viscosity with respect to the other tested electrolytes, it is a promising candidate for anode‐free batteries. Na‐EC/PC has only a limited reductive stability, which might cause low efficiency and severe electrolyte decomposition. LHCE suffers from high viscosity and slow ion kinetics, which might decrease its suitability for high current densities and low temperatures.

### 2.2 Na||C‐Al Half‐Cell Tests

To compare the plating/stripping mechanisms for the different electrolytes, half cells were cycled in two‐ and three‐electrode configurations and under varying current densities and temperatures.

**FIGURE 2:**
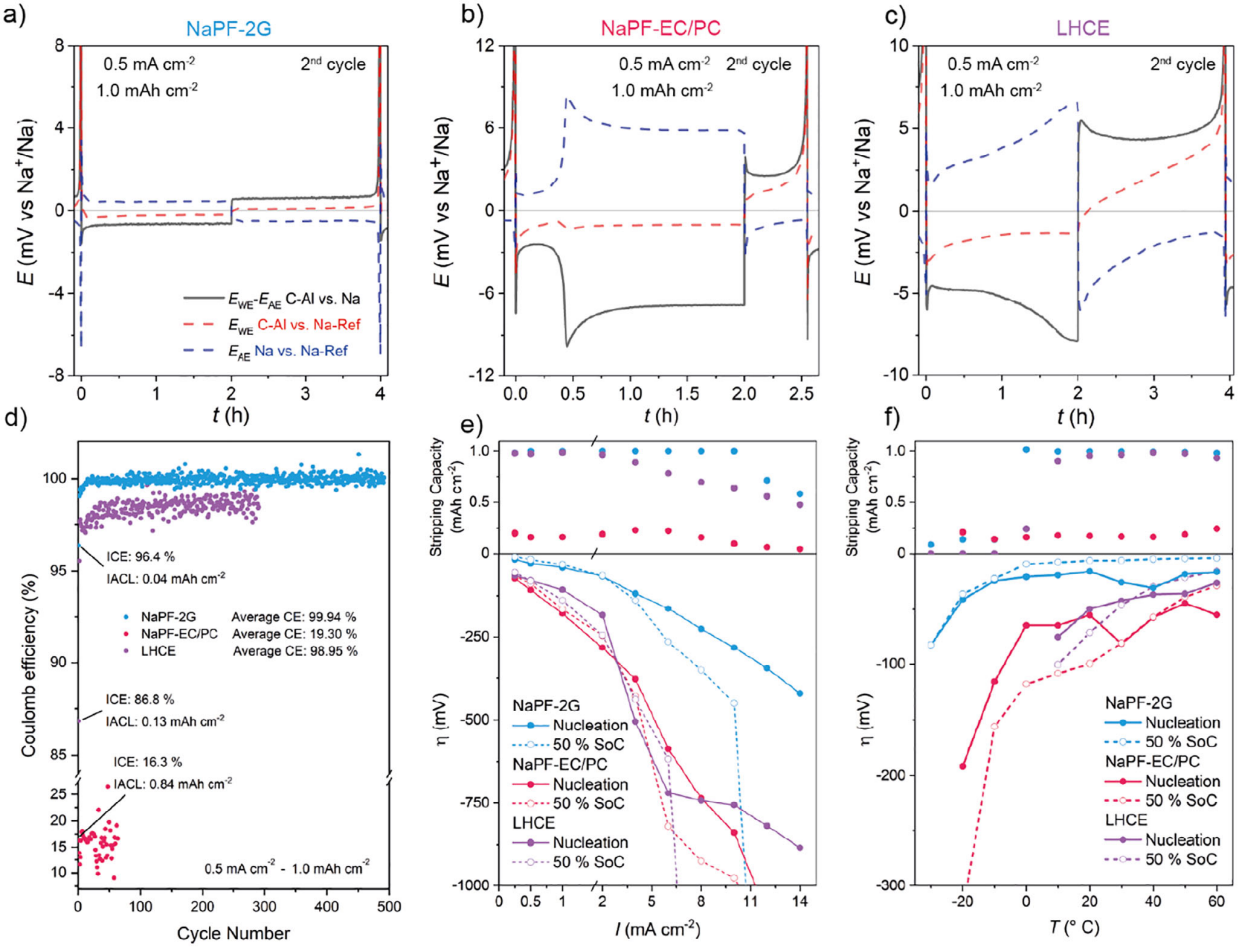
Plating/stripping behavior of Na in three‐electrode half‐cells with a) NaPF‐2G, b) NaPF‐EC/PC, and c) LHCE, at 0.5 mA cm^−2^ and 1.0 mAh cm^−2^ (second cycle). *E*_WE_, *E*_AE_, and *E*_RE_ are the potentials of the working (C‐Al foil), counter (Na), and reference electrode (Na). d) Shows the Coulomb efficiency in coin cells with the ICE and IACL. The stripping capacity and overpotential during nucleation and plating (50% SoC): e) with current densities ranging 0.25–14 mA cm^−2^ with 1.0 mAh cm^−2^ f) and temperatures from −30°C to 60°C in 10°C increments with 0.5 mA cm^−2^ and 1.0 mAh cm^−2^. The currents correspond to C‐rates ranging from 0.25 to 14 C relative to 1 mAh cm^−2^ as the full capacity.

The sodium plating/stripping behavior for the three electrolytes was evaluated in three‐electrode cells (Figure 2a–c). A carbon‐coated aluminum current collector (C‐Al) served as the working electrode (WE), while Na was used for the counter electrode (also called auxiliary electrode AE) and the reference electrode (RE). The commercial coated Al‐foil has a homogeneous carbon‐coating with a thickness of about 1 – 1.5 µm as determined by SEM (Figure S3). The density is defined as 0.5 g m^−2^ by the manufacturer. The Raman spectrum of the carbon coating showed a D Band to G Band ratio of 1.02, typical for disordered carbon (Figure S4a) [51]. Compared to the uncoated Al foil, nucleation and plating show lower overpotentials as seen in Figure S4b, indicating an increased sodiophilicity [16]. Moreover, the initial Coulomb efficiency (ICE) in NaPF‐2G with coating is 96% and without 82%, demonstrating reduced Na consumption in the first cycle, thus showing an initial areal capacity loss (IACL) of 0.04 and 0.18 mAh cm^−2^, respectively. The initial areal capacity loss is here introduced as a parameter to provide clearer information on the capacity loss that needs to be compensated in full cells by the cathode active material (CAM) or sacrificial salts, see Section 2.4. With the carbon coating, insertion of Na is visible in the first cycle between 0.3 and 0.0 V. *E*_WE_, *E*_AE_, and *E*_RE_ are the electrode potentials of the working, auxiliary, and reference electrodes, respectively. *E*_WE_−*E*_AE_ is the cell voltage.

As the first cycle exhibits higher overpotentials due to the SEI formation on the Na counter electrodes and effects such as dendrite stripping have not yet occurred, data interpretation is conducted for the second cycle. The initial cycle, along with further cycles, is shown in Figure S5.

For 2G, the initial plating overpotential (−6.2 mV) arises mainly from the Na counter electrode, while the nucleation on the C‐Al foil exhibits only a small overpotential in the range of −0.2 mV. Upon switching from plating to stripping on the C‐Al, the potential curves invert, but no significant overpotential is observed for the start of the stripping from the C‐Al. At the end of the stripping, the C‐Al electrode potential rises quickly until the cut‐off potential is reached, indicating a defined and complete removal of electroactive Na. Overall, the Na electrode shows around two times higher polarization than the C‐Al current collector during plating, likely due to a less ideal surface and a thicker SEI. In contrast, Na plated on C‐Al is reformed each cycle, indicating a low resistive and favorable interface.

In the EC/PC electrolyte, the C‐Al plating potential exhibits an almost stable plateau, while the Na electrode shows variations during stripping. At a lower overpotential, freshly deposited Na is stripped from the AE due to the thinner SEI, a process known as dendrite stripping. Once the dendrites are depleted, bulk Na stripping begins, which is hindered by the thicker SEI on the Na bulk compared to the dendrites, causing the potential rise and leading to a voltage maximum. The stripping of bulk material results in pit formation after the SEI on the surface is removed. The bulk material in the pit has a thinner, modified, or fractured SEI. The dissolution from the pits results in a decrease in cell voltage, and the voltage curve shows a plateau. Mandl et al. observed a similar behavior for Na symmetrical cells with carbonate‐based electrolytes [52]. The amount of dendrite stripping aligns well with the amount of plating that happened on the Na electrode in the preceding half‐cycle.

In the LHCE, for the first half‐cycle, the cell voltage curve shows a plateau after nucleation and afterward a slope of increasing overpotential. Looking at the individual potential curves, the overpotential at the WE eventually slowly decreases until reaching a plateau at around half of the plating step, indicating a change in plating resistance during the deposition. At the AE, the overpotential increases, indicating dendrite stripping. As the efficiency in LHCE is higher than in NaPF‐EC/PC and close to 100%, mainly dendrite stripping and no pit formation occur in LHCE, which would otherwise be indicated by a decrease of stripping overpotential at the Na AE. As seen in Figure S5, this effect mainly occurs in the first cycles. Overall, the plating overpotential at 0.5 mA cm^−2^ at the working electrode ranks NaPF‐2G (−0.2 mV) < NaPF‐EC/PC (−1.1 mV) < LHCE (−1.4 mV), indicating the best permeability for the Na ions to nucleate on the anode surface, and therefore the highest sodiophilicity, with 2G.

Long‐term cycling for up to 500 plating/stripping cycles was performed in two‐electrode coin cells. The CE versus cycle number is shown in Figure 2d. NaPF‐2G has an average CE of 99.94% for 400 cycles, NaPF‐EC/PC has an average CE of 19.30% over around 60 cycles, and LHCE shows an average CE of 98.95% after 300 cycles. The ICE decrease in the order NaPF‐2G (96.4%, 0.036 mAh cm^−2^) > LHCE (86.8%,) > NaPF‐EC/PC (16.3%). The IACL increases in the similar order NaPF‐2G (0.036 mAh cm^−2^) < LHCE (0.132 mAh cm^−2^) < NaPF‐EC/PC (0.837 mAh cm^−2^).; however, in metal‐based batteries, the CE does not scale with increasing areal loading, as shown in Figure S6, where areal capacities of 1 and 2 mAh cm^−2^ result in similar capacity loss but different efficiencies. Consequently, the absolute IACL is a more meaningful metric than relative percentage values, in contrast to intercalation or conversion‐type active material. The 2G‐based electrolyte performs very well, probably due to the beneficial thin SEI formed with NaPF‐2G. The poor performance of the carbonate‐based electrolyte was expected due to the weak reductive stability of the electrolyte against sodium metal, as seen in Figure 1d, and it is also reported in the literature [17]. Due to the low reductive stability, electrolyte decomposition happens during the Na deposition, leading to thick SEI and co‐deposition of electrolyte decomposition compounds with the Na. Therefore, Na is either consumed during electrolyte decomposition or becomes electrochemically isolated and is, therefore, no longer available. Although having similar reductive stability as NaPF‐2G, LHCE shows lower CE and seems to have a less beneficial Na deposition mechanism. The lower ICE of LHCE indicates that mainly in the beginning, severe electrolyte decomposition along with the isolation of Na metal occurs, caused by a weaker electrolyte stability than NaPF‐2G.

To evaluate the rate capability of the Na plating/stripping, coin cells were cycled with the three electrolytes at increasing current densities ranging from 0.25 to 14 mA cm^−2^, while maintaining the areal capacity of 1 mAh cm^−2^. This theoretically corresponds to C‐rates from 0.25 to 14 C relative to 1 mAh cm^−2^ as the full capacity. Besides the stripping capacity, the graph shows the nucleation overpotential at the beginning of the plating step and the overpotential at 50% state of charge (SoC) during further plating, see Figure 2e. The CE and individual potential curves are shown in Figures S7 and S9a. The nucleation potentials on the C‐Al current collector at a current density of 0.5 mA cm^−2^ in coin cells increase in the order NaPF‐2G (30 mV) < LHCE (80 mV) < EC/PC (100 mV). The overpotential at 50% SoC increases in the order NaPF‐2G (15 mV) < LHCE (80 mV) < EC/PC (90 mV).

NaPF‐2G exhibits excellent rate capability, sustaining cycling to 8 mA cm^−2^. As expected, the overpotential of the plating increases with increasing current density. At 10 mA cm^−2^ and above, the capacity decreases as the overpotential during the plating/stripping increases drastically and exceeds the voltage cut‐off point of 2 V (see Figure 2e). Under these conditions, the ion transport becomes insufficient to sustain the continuous Na‐ion flux from the AE to the WE. This results in Na‐ion depletion at the plating surface, which increases the overpotential until the cut‐off potential is reached. A similar phenomenon was observed by Bai et al. for the plating with Li [53].

In solid‐state Na anode‐free cells, overpotentials of around 100 mV for the initial nucleation and around 40 mV for the deposition are observed at current densities of 0.5 mA cm^−2^, which is in the same region as it is found for the ether‐based electrolyte at similar current densities [54, 55] The plating/stripping performance with the EC/PC‐based electrolyte is poor and deteriorates even more at current densities above 6 mA cm^−2^, as expected from the limited reductive stability of EC/PC observed in the ESW measurements and poor performance in long‐term measurements (see Figures 1d and 2d).

For LHCE, at a current density of 8 mA cm^−2^, the cell cannot plate the full 1 mAh cm^−2^ because the deposition overpotential increases above the cut‐off of 2.0 V, although nucleation is still possible at a lower overpotential (Figures S7 and S9a). With increasing current density, the resistance of the electrolyte increases. As the LHCE already has a higher resistance due to the high viscosity and low ion conductivity see Figure 1a,b, the ion mobility decreases further with higher current density. This results in a lower rate capability for LHCE compared to the 2G‐based electrolyte. As seen in Figure 2c, the LHCE cells need more cycles to reach a stable CE compared to NaPF‐2G. Therefore, LHCE cells were cycled ten times at 0.5 mA cm^−2^ for 1 mAh cm^−2^ before the rate capability test to reach significantly better rate capability.

In addition to testing room‐temperature cells, cycling at different temperatures provides insights into the evolution of nucleation processes and charge transport within the electrolyte and across the SEI [49]. As shown in Figure 2f, Figures S8, and S9b, for 2G, the nucleation barrier and overpotential at 50% SOC increase markedly below 0°C; nevertheless, 2G still outperforms the other two electrolytes under these conditions. This limitation can be attributed to the reduced ionic conductivity of the 2G electrolyte at low temperatures, as previously discussed. The abrupt change below 10°C aligns with the hysteresis observed in the conductivity measurements, suggesting a structural transformation in the electrolyte, even though no transition was detected by DSC. In contrast, for EC/PC, a consistently low capacity across the entire temperature range is observed. For LHCE, the full capacity of 1 mAh cm^−2^ can only be reached above 0°C; at lower temperatures, the high viscosity and correspondingly low ionic conductivity impose severe restrictions on ion transport.

### 2.3 Operando Optical Microscopy and Further Characterization of the Plating Morphology and SEI

Operando optical microscopy was employed to study the morphological changes during plating and stripping. The experimental setup is shown in Figure S10. The operando cells were cycled at 0.25 mA cm^−2^ with a capacity of 1 mAh cm^−2^.

**FIGURE 3:**
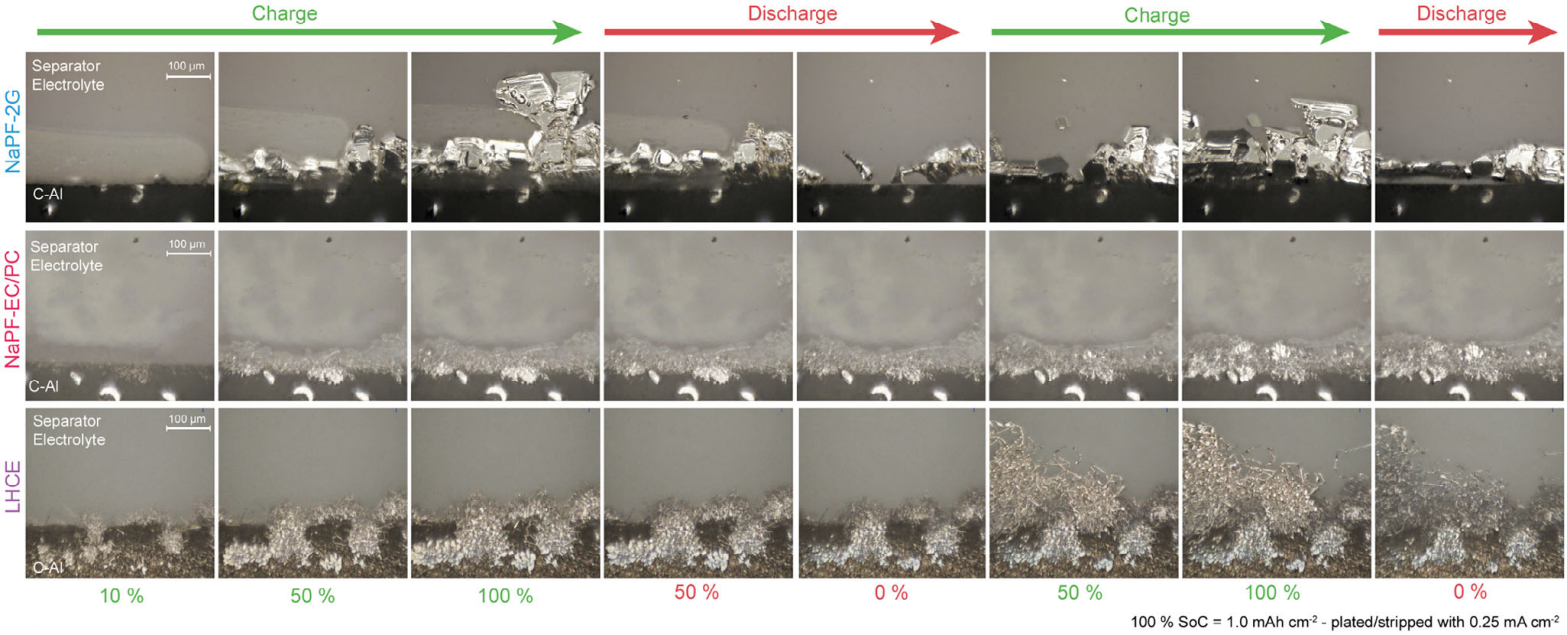
Operando optical microscopy results for NaPF‐2G (top), NaPF‐EC/PC (middle), and LHCE (bottom). The cells were cycled in an optical cell at 0.25 mA cm^−2^ with 1 mAh cm^−2^ capacity. The deposition and dissolution of Na on the edge of a C‐Al working electrode were observed.

With 2G, the onset of plating is marked by the appearance of nucleation sites, visible as shiny silver regions of Na metal on the edge of a dark C‐Al background (Figure 3; Video S1a). At 10% SoC, only a few relatively large nucleation sites are present, leaving much of the C‐Al surface uncovered. Plating proceeds from these initial sites, forming shiny, geometrically ordered, crystal‐like Na deposits, with one structure extending further into the electrolyte. In addition, the appearance of a gas bubble, visible as a light grey round sphere at the edge of the electrode, indicates decomposition during the initial plating step [56]. Upon stripping, the Na dissolves uniformly from the electrode surface, although some Na metal remains behind. The CE of the first plating/stripping cycle in the operando setup is 86%. The relatively large amount of isolated Na observed in the operando cell likely arises from the lack of pressure at the electrode edges, the diverging electric field near the electrode boundary, and the inferior sealing of the operando setup compared to coin cells. These factors promote preferential deposition and incomplete stripping at the electrode edges [57].

During the second plating step, at the onset of plating, the gap between the current collector and isolated Na is rapidly filled, after which Na continues to grow outward from the previously formed structures. This indicates that growth on pre‐existing Na is energetically more favorable than direct nucleation on the C‐Al surface. The crystal‐like morphology is preserved as plating progresses.

Additionally, the operando cells reveal the decoupling of Na fragments during plating/stripping, followed by their movement and reconnection with bulk Na, as previously reported for Li by Liu et al. [58]. Similar shiny Na deposits in 2G were observed in our experiments and have also been reported by Ma et al. [18]. We attribute the superficial plating behavior to a nanometer‐thin SEI, which allows facile transport of Na‐ions, enabling a step‐flowing growth mechanism. Unlike Li, the SEI on Na does not significantly hinder metal deposition on the surface.

The coin cell experiments showed particularly poor plating/stripping performance for the EC/PC electrolyte (Figure 2d), which is confirmed by operando optical microscopy. During the initial plating, very fine, mossy Na structures are clearly visible (Figure 3; Video S1b). Nucleation occurs at many sites simultaneously, but unlike 2G, these sites do not develop into bulk crystals that can cover large areas. Instead, each nucleation site is quickly passivated, as reflected by the limited shiny appearance, indicating more parasitic side reactions and the formation of a thicker SEI. As a result, new nucleation sites continuously form, producing numerous small structures with a large total surface area and accelerating electrolyte decomposition. Bulk crystal growth occurs only in a few isolated regions. During stripping, only a small fraction of the plated Na is removed, while large amounts remain as isolated or reacted Na, which accumulate over subsequent cycles. Gas bubbles forming around the plated Na further indicate electrolyte decomposition during plating [56]. In the following cycles, Na plating initiates primarily from the current collector rather than on top of the previously isolated Na [59].

The LHCE is dominated by bush‐like Na structure in the first cycle, with some larger particle agglomerations within, growing towards the electrolyte and partially covering the C‐Al surface (Figure 3; Video S1c) [60]. These structures appear shinier than in EC/PC; nevertheless, they indicate parasitic side reactions with the electrolyte compared to 2G. While stripping, most of the structures remain, explaining the low ICE in the coin cell tests. In the second cycle, when SEI is less dominant, the growth mechanism becomes more apparent (Video S1c). Initially, thin shiny Na whiskers rapidly grow from their roots rather than their surface. Around 50% SOC, the mechanism changes: round, pearl‐like structures appear on the whiskers, coinciding with an increase in overpotential. Similar growth patterns have been observed for Li plating, though Na whiskers tend to be larger and more stable [48].

The growth mechanism in the LHCE electrolyte can be described in three steps. First, nucleation occurs beneath the existing SEI, which is permeable to Na‐ion. In the second step, nucleated Na grows and eventually breaks through the SEI. Freshly exposed metal immediately forms a secondary SEI, which inhibits further plating on the surface. Consequently, subsequent metal deposition continues from the root, where the electrode SEI is easier to penetrate. During whisker growth and displacement toward the separator, the secondary SEI experiences mechanical stress, particularly at kinks, causing local thinning. In the third step, plating may shift from root growth to surface growth at these weakened SEI regions [48]. This also aligns with the slow decrease of overpotential followed by a plateau for the plating on C‐Al in LHCE, as observed in Figure 2c, as the surface growth is expected to have a lower overpotential than the root growth. The resulting whiskers are generally not straight needles but form loops. Some early‐formed needles connect at their tips to create loops, and growth can also originate from kinks in existing whiskers [60].

Several effects must be considered when interpreting the results. Images are taken at the edge of the electrode, where so‐called edge effects can occur due to the non‐uniform electric field compared to the bulk electrode surface [57]. Moreover, unlike the bulk electrode, no pressure is exerted in the axial direction at the edges. Since mechanical pressure within the cell is known to strongly influence the plating morphology, these factors may influence local deposition [61]. Nevertheless, comparison of their plating and stripping behavior remains meaningful. So far, most operando microscopy studies have been performed in capillary cells or other operando cell conformations that had a large distance between the electrodes, which is only filled with electrolyte [18, 58]. The large distance and absence of any separator and pressure greatly affect the cell resistance, ion distribution, and consequently plating morphologies, benefiting the growth of large dendritic structures. While in our cell design, the distance between the electrodes is as short as in a coin cell, and a separator is pressed against the plating surface at least in the vertical direction.

**FIGURE 4:**
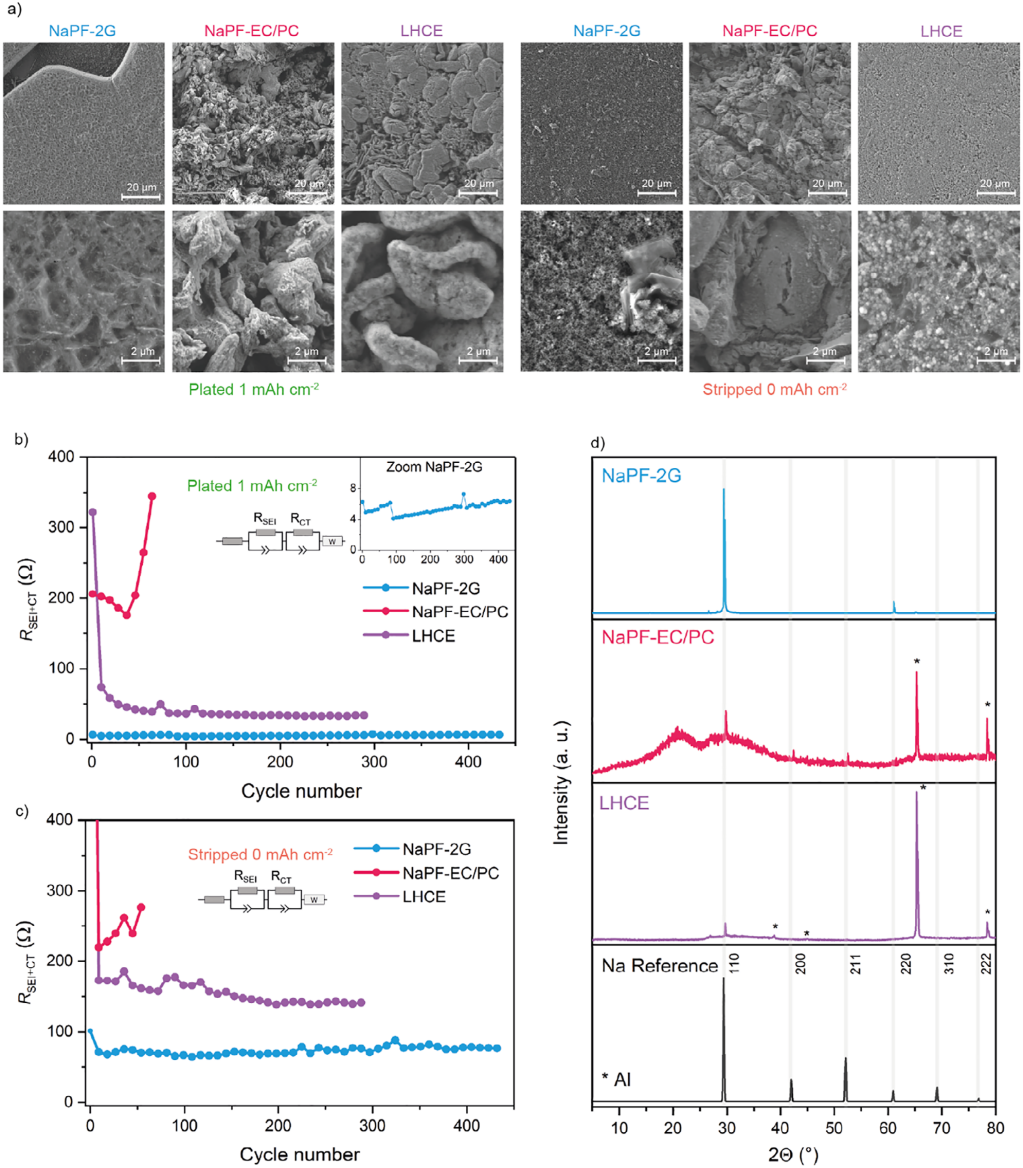
Characterization of Na deposited on the C‐Al current collector for NaPF‐2G (blue), NaPF‐EC/PC (red), LHCE (violet): a) SEM micrographs after three cycles of plating/stripping (1 mAh cm^−2^, 0.25 mA cm^−2^). Impedance of the SEI and charge transfer processes plotted over the cycle number of C‐Al vs. Na coin cells in b) plated and c) stripped state cycled at 0.5 mA cm^−2^ and 1.0 mAh cm^−2^. d) XRD patterns of 5.0 mAh cm^−2^ Na deposited on C‐Al in the three electrolytes compared to Na metal reference.

To cross‐check the observed morphologies from the optical microscopy, scanning electron microscopy (SEM) was performed with ex situ received electrodes from coin cells after three cycles of plating and stripping, respectively, see Figure 4a. Different from the operando microscopy measurement, for the SEM, the images are taken from the bulk electrode surface, so pressure was applied during plating and stripping, and also, the edge effect does not come into play in the observed areas. Nonetheless, similarly looking Na blocks can be seen for plating in 2G. In the SEM, one can also see that the surface of the Na plated in 2G is not completely smooth; instead, it shows a network‐like structure. But one needs to consider that Na melts quickly under the ion beam of conventional SEM, and similar network structures were also observed with plated Li due to beam damage [62]. To prevent beam damage, cryo‐SEM may need to be applied. Also, ex situ SEM pictures of stripped C‐Al electrodes in 2G show that almost no isolated Na is left in coin cells; only the fine structure of the carbon coating is visible. The SEM images of Na plated in EC/PC show the fine and very inhomogeneous plating. Similar to optical microscopy, the plated and stripped states look almost identical. In the SEM images of LHCE plating, one can see a mix of whiskers and finer round particles. One also sees that the particles have small cracks or pores on the surface, which affirms that the SEI is breaking to allow Na stripping from the inside. Overall, the observations from optical microscopy align well with the SEM images of the planar electrode surfaces.

Impedance spectra were recorded under the same cycling conditions as in Figure 2d for the different electrolytes in two‐electrode coin cells. A reference spectrum was first collected from the pristine electrodes before cycling. Thereafter, spectra were taken after the first plating half‐cycle and subsequently every ninth cycle, see Figure 4b,c. Measurements were conducted both before and after plating, which allows for comparison of charge transfer on sodium metal and C‐Al.

The Nyquist plots show two semicircles and a Warburg element (Figure S11), fitted as SEI and charge‐transfer resistance. Since the spectra of the three electrolytes differ and the limited frequency range (100 mHz–1 kHz) complicates separation, both semicircles were combined into a single interfacial resistance plotted in Figure 4b,c [11].

In the plated state (Figure 4b), deposition in NaPF‐2G exhibits a resistance below 10 Ω, increasing only slightly over 400 cycles, demonstrating excellent SEI stability. In EC/PC, resistance starts at ∼206 Ω and drops to ∼175 Ω by cycle 40, reflecting an increase in surface area as seen in optical microscopy, but then rises rapidly, signaling excessive electrolyte decomposition and unstable cycling. LHCE initially shows a high resistance (∼320 Ω) in the first cycle, which drops below 50 Ω in subsequent cycles and gradually decreases toward 30 Ω. This early behavior reflects SEI formation and electrolyte consumption, supporting root‐growth plating and low initial CE.

For the stripped/pristine state, 2G shows the lowest resistance at ∼70 Ω, with the pristine C‐Al at ∼100 Ω, indicating favorable nucleation on C‐Al in 2G. The resistance is very stable over time and increases slightly. EC/PC exhibits a high pristine resistance of 1175 Ω, which drops to ∼220 Ω after the first cycle—reflecting initial SEI formation—but then rises continuously until cell failure, consistent with optical microscopy and SEM observations of isolated Na accumulating on the electrode. LHCE shows an even higher initial resistance of 1938 Ω, which drops below 200 Ω during cycling and slowly decreases further. The high initial resistance corresponds to the large nucleation overpotential (∼0.5 V) observed in the first plating cycle (Figure 2c). Therefore, the SEI formed on the C‐Al also varies in thickness between the three electrolytes. The differences in resistance between stripped and plated states for 2G and LHCE suggest that surface plating on existing Na is more favorable than nucleation on the current collector, or that a beneficial interphase forms on the C‐Al. Additionally, impedance was measured in three‐electrode PAT cells with shorter runtime to observe the impedance between C‐Al WE and a Na RE independently of the Na AE. Similar trends for the development of resistance on the electrode can be observed as in the two‐electrode cells (Figures S12 and S13).

Optical microscopy revealed that Na deposition in a 2G electrolyte exhibits a highly ordered, crystal‐like morphology. Ex situ XRD (Figure 4d) further confirms this remarkable behavior, showing a strong preferred orientation along the 110 (and 220) axes, while other reflections are absent. This indicates that C‐Al with 2G enables uniquely highly crystalline Na plating, a phenomenon not commonly observed in sodium or even lithium metal systems. In contrast, EC/PC shows only weak signals for 110, 200, and 211, indicating predominantly amorphous deposition with low crystallinity. LHCE exhibits intermediate crystallinity, with only the 110 and 222 reflections detectable. Also, in solid‐state Na anode‐free cells, crystalline microstructures with varying orientations could be observed, while the crystallinity in NaPF‐2G is dominated by one orientation [55]. The results highlight that 2G uniquely promotes exceptional electrochemical growth of crystals, which directly correlates with its high reversibility and stable cycling performance.

### 2.4 Anode‐Free Full Cells with Na_4_Fe_3_(PO_4_)_2_(P_2_O_7_) as CAM

To illustrate the superior performance of the 2G‐based electrolytes for anode‐free SIBs also on a macroscopic level, a full cell with Na_4_Fe_3_(PO_4_)_2_(P_2_O_7_) (NFPP) as CAM was assembled (C‐Al│NFPP). NFPP is a very low‐cost CAM that is based on abundant elements only. It has a theoretical specific capacity of 129 mAh g^−1^ and shows an average redox potential of 3.1 V vs. Na^+^/Na that is compatible with glyme‐based electrolytes [63, 64, 65].

**FIGURE 5:**
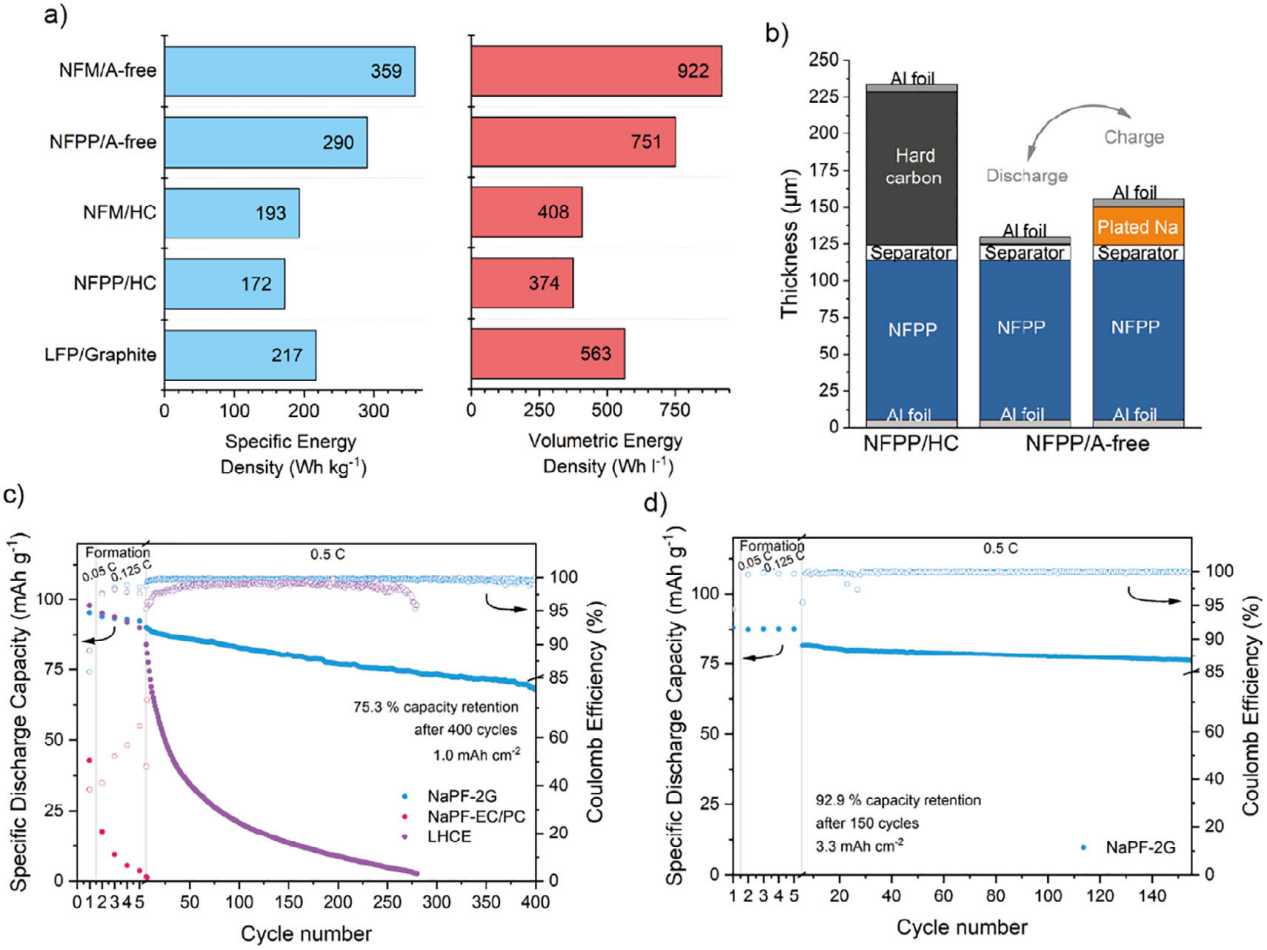
Full cell results and  a) comparison in energy densities for several SIB cell concepts and a Li‐ion battery benchmark assuming double‐side coated stack unit. *Anode*: Anode‐free (A‐free), hard carbon (HC), or Graphite. *Cathode*: NaNi_1/3_Fe_1/3_Mn_1/3_O_2_ (NFM), Na_4_Fe_3_(PO_4_)_2_(P_2_O_7_) (NFPP), LiFePO_4_ (LFP). b) Comparison of cell stack thickness for a Na‐ion cell with anode‐free design and with hard carbon as anode (minimum repetition unit). c) Full‐cell cycling of NFPP|C‐Al for the electrolytes NaPF‐2G (blue), NaPF‐EC/PC (red), LHCE (violet) at 0.5 C after formation, showing the specific discharge capacity of NFPP and the Coulomb efficiency over cycling. As no excess Na metal was used during cell assembly, i.e., the anode‐free design is also referred to as “zero excess”. d) Full‐cell cycling of NFPP|C‐Al for the electrolyte NaPF‐2G (blue) with a capacity loading of 3.3 mAh cm^−2^.

On the materials basis (weight of CAM only), the theoretical energy densities of anode‐free cells with NFPP as CAM are 400 Wh kg^−1^ and 1293 Wh l^−1^. While a technical optimization of the cells is outside the scope of our study, the achievable energy densities of an anode‐free cell with NFPP as CAM was evaluated and compared to state‐of‐the art Na‐ion and Li‐ion (LFP) chemistries, see Figure 5a. For a more realistic view, specific energies were calculated for a minimum stack unit consisting of: one double‐side coated cathode (C), one double‐side coated anode (A), and two separator layers (S) arranged according C/S/A/S. Composite porosity has been set to 25%, 35%, and 50% for cathode, anode, and separator, respectively. A cathode areal capacity of 3 mAh cm^−2^ was balanced with an anode loading of 3.3 mAh cm^−2^ for cells with hard carbon and graphite (A/C‐ratio = 1.1). Electrolyte weight was considered assuming the porosity of the stack being completely filled. Current collectors weight was added for 10 µm thick Cu and Al foils (depending on whether SIB or LIB are considered). All values used for calculation are given in Tables S3 and S4.

The energy densities for an anode‐free SIB design with NFPP as CAM could reach values of 290 Wh kg^−1^ and 751 Wh L^−1^ being significantly higher compared to a conventional Na‐ion cell design containing NFPP as CAM and hard carbon as AAM (172 Wh kg^−1^ and 374 Wh L^−1^), see Figure 5a. The comparison also illustrates the significant benefit in volumetric energy density when transitioning to the anode‐less design. This difference can also be clearly seen in the comparison of the stack thicknesses (0.5x C/S/A/S) between anode‐free NFPP design and conventional SIBs with NFPP and hard carbon in Figure 5b. Values are also higher compared to a Li‐ion cell with LiFePO_4_ (LFP) as CAM and Graphite as AAM (217 Wh kg^−1^ and 563 Wh l^−1^). Even higher values could be obtained for anode‐free designs with layered oxides (NFM) as CAM, however, the use of ether solvents may be limited in this case due to the higher cell voltage. The analysis clearly shows that very attractive energy densities could be reached with the anode‐less design, even for the low‐cost NFPP as CAM, and that the concept is one of the few strategies for even exceeding the often‐mentioned Li‐ion LFP benchmark.

To test the concept, anode‐free Na/NFPP cells with the different electrolytes were assembled and cycled for up to 400 cycles. Initial cycles were done at low rates (C/20, C/8) followed by continuous cycling at C/2 (0.5 mA cm^−2^). The areal capacity of 1 mAh cm^−2^ was chosen to ensure comparability with the previous half‐cell tests. As shown in Figure 5c, NaPF‐2G enabled an initial discharge capacity of 96.14 mAh g^−1^, an ICE of 85.6%, and an initial areal capacity loss (IACL) of 0.14 mAh cm^−2^. After 400 cycles the cell retained 75.8% of the initial discharge capacity after formation with an average CE of 99.68%. These results are clearly superior compared to the other electrolytes. LHCE exhibited an average CE of 96.06%, while EC/PC showed only 55.22%. Due to the limited sodium reservoir in the zero‐excess setup, capacities in EC/PC and LHCE dropped rapidly: in EC/PC, capacity retention fell to 42% by the second cycle; in LHCE, retention dropped below 80% after eight cycles. These results confirm the compatibility of glyme‐based electrolytes for anode‐free SIBs. We also prepared cells with higher NFPP areal loadings of 3.3 mAh cm^−2^ which better align with commercial requirements. The high‐loading NFPP cathodes allow stable long‐term cycling, achieving a capacity retention of 92.9% after 150 cycles (Figure 5d). In half‐cell setups, reversible Na plating/stripping was observed at areal capacities of 10 mAh cm^−2^, indicating that the high areal loadings are not intrinsically limited by the anode‐free configuration (Figure S14).

In the initial cycle, the 1.0 mAh cm^−2^ full cell showed an IACL of 0.14 mAh cm^−2^ while in the high loading 3.3 mAh cm^−2^ cell, the capacity loss in the initial cycle is 0.16 mAh cm^−2^. This indicates that the initial capacity loss is constant with the surface area of the anode current collector and does not scale up with higher mass loadings, as stated also in the half‐cell measurements, see chapter 2.2. In contrast, the cathode‐related losses scale with higher loadings, explaining the marginally increased IACL. Therefore, a specific capacity amount is lost due to initial SEI formation on the surface of the anode current collector. In practical cells, this amount needs to be compensated by the cathode active material. In the present case for the NFPP cathode with 3.3 mAh cm^−2^, the IACL loss of 0.16 mAh cm^−2^ amounts to only 4.8%. As discussed above, significantly lower losses of approximately 0.04 mAh cm^−^^2^ were observed in half‐cell measurements at 1 and 2 mAh cm^−^^2^, which can be attributed to the absence of cathode‐related irreversible losses in these configurations.

Overall, to the best of our knowledge, we demonstrate for the first time a zero‐excess, anode‐free full cell employing high mass loading and low‐cost, with already commercially available materials.

Finally, it should be noted that the IACL in anode‐free cell designs is ideally a constant value. This contrasts with Na‐ion cells employing conventional anodes, such as hard carbon. In those systems, the IACL increases with areal loading; in other words, the thicker the electrode, the greater the Na loss due to SEI formation, and consequently, the larger the IACL.

## 3 Conclusion

Sodium plating and stripping were studied for three different electrolytes based on carbonates (EC/PC), ether (diglyme, 2G), and for a localized high‐concentration electrolyte (LHCE, 1G/TTE). A commercial carbon coated Al‐current collector was used in all cases with the target to develop anode‐free SIBs. Operando optical microscopy and electrochemical analysis revealed a strong electrolyte dependence on the morphology and reversibility of the plated sodium. The 2G electrolyte showed clearly superior performance, producing highly crystalline and uniform sodium deposits. Its low viscosity, high ionic conductivity, and reductive stability enable a thin, low‐resistive SEI and nearly loss‐free plating (average CE 99.94%), with kinetic limitations only below 0°C or above 10 mA cm^−^^2^. This defined Na plating, confirmed by optical microscopy and XRD, has not been reported for lithium. In contrast, EC/PC decomposes rapidly due to poor reductive stability, forming mossy Na and thick, more resistive SEI layers, while LHCE suffers from high viscosity, limited ion mobility, and high impedance in the first cycles, leading to whisker‐type growth. Further, we introduce the IACL as relevant parameter for discussing the performance of anode−free cell designs. The IACL quantifies the additional CAM areal capacity required to compensate for the Na loss occurring during the initial Na plating process. In anode−free cells, the IACL is ideally a constant value which does not change with the cathode loading. For thick cathode loadings, the IACL therefore can become negligible. This contrasts with Na−ion batteries containing conventional anode active materials such as hard carbon for which the IACL increases with the amount of anode material. Therefore, we propose comparing anode−free cells using the IACL rather than the initial Coulomb efficiency (ICE). The applicability of the electrolytes was further tested in full cells (anode‐free design) with NFPP as CAM and a high mass loading above 3 mAh cm^−2^. Benchmarking of the approach against state‐of‐the art Na‐ion and Li‐ion (LFP) shows the attractiveness of the approach. The clearly best results were obtained for the 2G‐based electrolyte, which enabled stable cycling with a capacity retention of 75.3% after 400 cycles. While further improvements may be possible by further optimizing current collectors and electrolyte formulations, the results underline the use of ether‐based electrolytes as the benchmark for anode‐free SIB.

Importantly, experiments were conducted using readily available, cost‐effective materials rather than expensive components, including carbon‐coated current collectors, diglyme‐based solvents, NaPF_6_ salt, and NFPP cathodes. This highlights that promising performance in anode‐free SIBs might already be achieved using commercially accessible components.

## 4 Experimental Section/Methods

### 4.1 Electrode and Cell Preparation, Cell Cycling

The working electrodes consisted of carbon‐coated aluminum current collectors (MTI Corp.), which were punched into electrodes and dried under vacuum overnight at 110°C. For the Na_4_Fe_3_(PO_4_)_2_(P_2_O_7_) (NFPP) electrodes, 94% NFPP, 3% PVDF, and 3% C65 were mixed with NMP into a slurry and coated on carbon‐coated Al foil under air. After calendaring at 140°C, circular electrodes of 12 mm diameter were punched and dried under vacuum overnight at 110°C. For galvanostatic cycling, CR2032 coin cells (MTI Corp.) and three‐electrode PAT cells (EL‐Cell) were assembled with Na electrodes of 12 mm diameter. To avoid sodium plating on the coin cell housing, an oversized C‐Al working electrode with 16 mm diameter was used. For the area capacity, the surface area of the Na electrode is taken. Three‐electrode Swagelok cell‐type cells were assembled with electrodes of 12 mm diameter. The cell assembly was performed in an argon‐filled glovebox from MBraun (H2O < 0.1 ppm, O2 < 0.1 ppm). Na metal (BASF) was used as the counter electrode of 12 mm diameter, and a Whatman membrane (GF/A) and a Celgard 2325 membrane (not for EC/PC) as the separators with a volume of 100 µL of electrolyte. The PAT cells use a specific Na metal ring (EL‐Cell) as the reference electrode.

For the electrolyte preparation, Sodium Bis(fluorosulfonyl)imide (NaFSI, purity 99.5%, Solvionic) was used as salt. Monoglyme (G1, Sigma–Aldrich) and 1,1,2,2‐Tetrafluoroethyl 2,2,3,3‐tetrafluoropropylether (TTE, Apollo Scientific) were pre‐dried with 4 Å porous molecular sieves overnight. The electrolytes 1 m NaPF_6_ in 2G and 1 m NaPF_6_ in EC/PC were received from E‐lyte.

The electrochemical galvanostatic charge–discharge experiments were conducted using an MPG 3 battery cycler from Biologic at the desired current. Cyclic voltammetry (CV) was performed in Swagelok‐type cells with 0.1 mV s^−1^ between −0.5 to 6.0 V.

For long‐term measurements in half‐cells, the cells were cycled five times at 5 mA cm^−2^ between 0 and 1 V to remove surface contamination [17]. Afterward, the cells were cycled at 0.5 mA cm^−2^ until a plated capacity of 1 mAh cm^−2^ was reached. The plated Na was stripped till a cut‐off voltage of 0.3 V.

### 4.2 Experimental Methods

Raman spectroscopy measurements were recorded with a Renishaw inVia confocal Raman microscope, using a 532 nm wavelength laser. For viscosity measurements, an IKA Rotavisc lo‐vi with a rotation of 100 rpm was used. For the conductivity measurement, a Biologic ITS‐e climate chamber with the HTCC conductivity cell with two platinum electrodes was used. The resistance was measured by EIS in the range of 1 MHz to 1 Hz. Differential Scanning Calorimetry (DSC) was performed on a METTLER TOLEDO Thermal Analysis System DSC 3 with a 40 µL standard Al crucible holder in a measurement environment of Ar 30 mL/min.

All NMR measurements were performed on a 400 MHz spectrometer fitted with a variable temperature unit using a Diff50 PFG‐NMR probe. Measurements were performed at room temperature. A stimulated echo gradient pulse sequence was used with the gradient length δ fixed at 1 ms and the diffusion time Δ set between 5 and 100 ms. The gradient steps were kept constant at 16. Recycle delays were set between 0.2 and 1.0 s.

For the operando optical microscopy, an EL‐Cell ECC Opto‐10 cell holder with a two‐electrode face‐to‐face arrangement of the carbon‐coated Al electrode and metallic counter electrode was used. A circular 12 mm diameter C‐Al electrode was folded in the middle to receive a smooth edge to observe the plating/stripping. The cells were cycled at 0.25 mA cm^−2^ for 1 mAh cm^−2^. A digital optical microscope (Keyence VHX‐7000) with a 500x magnification was used, and an image was taken every 120 s.

Scanning electron microscopy was performed on a Zeiss Merlin Gemini II at 5 kV and 500 pA using an SE2 detector and a Semilab sample transfer shuttle.

Electrochemical impedance spectroscopy (EIS) experiments were carried out to determine the resistance formation during the plating/stripping process. The electrochemical setup was prepared in a similar way to that described before. The coin cells and PAT cells were probed via EIS using a potentiostat (MPG‐3 from Biologic) in the frequency range of 100 mHz – 1 kHz.

X‐ray diffraction (XRD) was performed with a Bruker XRD Phaser D2 with an airtight sample holder.

## Conflicts of Interest

The authors declare no conflicts of interest.

## Supporting information




**Supporting File 1**: advs74455‐sup‐0001‐SuppMat.docx

**Supporting File 2**: advs74455‐sup‐0002‐Video S1.mp4

## Data Availability

The data that support the findings of this study are available from the corresponding author upon reasonable request.
