# Supplementary material for: Electrolyte‐Dependent Sodium Plating for Anode‐Free Na‐Ion Batteries Studied by Operando Optical Microscopy
Source: Adv Sci (Weinh). 2026 Feb 20;13(24):e00058. doi: 10.1002/advs.202600058 (PMC13115960; doi:10.1002/advs.202600058)
Supplement: Supplementary file 1 — Supporting File 1: advs74455‐sup‐0001‐SuppMat.docx [file ADVS-13-e00058-s002.docx]

Supporting information on

Electrolyte-Dependent Sodium Plating for Anode-Free Na-Ion Batteries Studied by Operando Optical Microscopy

Moritz Exner^1,3^, Dominik Stepien^1,3*^, Annica I. Freytag^1,3^, Pedro B. Groszewic^4,5^, Xiangping Min^1^, Nour Adrah^1^, Peter Axmann^6^, Philipp Adelhelm^1,2,3^*

^1^Institut für Chemie, Humboldt Universität zu Berlin, Brook-Taylor-Str. 2, 12489 Berlin, Germany
E-mail: [philipp.adelhelm@hu-berlin.de](mailto:philipp.adelhelm@hu-berlin.de), [dominik.stepien.2@hu-berlin.de](mailto:dominik.stepien.2@hu-berlin.de)

^2^Center for the Science of Materials Berlin (CSMB), Humboldt-Universität zu Berlin
Zum Großen Windkanal 2, 12489 Berlin, Germany

^3^Joint Research Group Operando Battery Analysis (CE-GOBA), Helmholtz-Zentrum Berlin für Materialien und Energie, Hahn-Meitner-Platz 1, 14109 Berlin, Germany

^4^ Department Spins in Energy Conversion and Quantum Information Science (SE-ASPIN), Helmholtz-Zentrum Berlin für Materialien und Energie, Hahn-Meitner-Platz 1, 14109 Berlin, Germany

^5^ Department of Radiation Science and Technology, Delft University of Technology, Mekelweg 15, Delft, 2629 JB, The Netherlands

^6^ ZSW Center for Solar Energy and Hydrogen Research Baden-Württemberg, Lise-Meitner-Straße 24, 89081 Ulm, Germany

*Correspondence: [philipp.adelhelm@hu-berlin.de](mailto:philipp.adelhelm@hu-berlin.de), [dominik.stepien.2@hu-berlin.de](mailto:dominik.stepien.2@hu-berlin.de)

**Figure S1**. Raman spectra of the electrolytes NaPF-2G (blue), NaPF-EC/PC (red), and LHCE (violet), including pristine measurements of the salt and solvents.

**Table S1**. VFT-fitting parameter of the viscosity results.

|  | **Viscosity fitting parameters** | | | | | | | |
| --- | --- | --- | --- | --- | --- | --- | --- | --- |
| solvent | *T*_0_  (K) | | *B*  (K) | | *η*_0_  x10^3^ | | E_A_  (kJ mol^-1^) | |
| 1M NaPF_6_ in 2G | 168 | ± 12 | 356 | ± 72 | 196 | ± 56 | 3.0 | ± 0.6 |
| 1M NaPF6 in EC/PC | 172 | ± 5 | 408 | ± 35 | 234 | ± 34 | 3.4 | ± 0.3 |
| NaFSI in 1G/TTE | 173 | ± 5 | 482 | ± 38 | 203 | ± 32 | 4.0 | ± 0.3 |

**Table S2.** VFT-fitting parameter of the conductivity results.

|  | **Conductivity fitting parameters** | | | | | | | |
| --- | --- | --- | --- | --- | --- | --- | --- | --- |
| solvent | *T*_0_  (K) | | *B*  (K) | | *σ*_0_ | | E_A_  (kJ mol^-1^) | |
| 1M NaPF_6_ in 2G | 198.0 | ± 2.1 | 149.7 | ± 6.0 | 31.9 | ± 0.9 | 1.24 | ± 0.05 |
| Cell 1M NaPF6 in EC/PC | 178.8 | ± 0.2 | 356.3 | ± 1.3 | 132.7 | ± 0.7 | 2.96 | ± 0.01 |
| NaFSI in 1G/TTE | 160.8 | ± 2.6 | 599.0 | ± 20.9 | 158.2 | ± 11.0 | 4.98 | ± 0.17 |


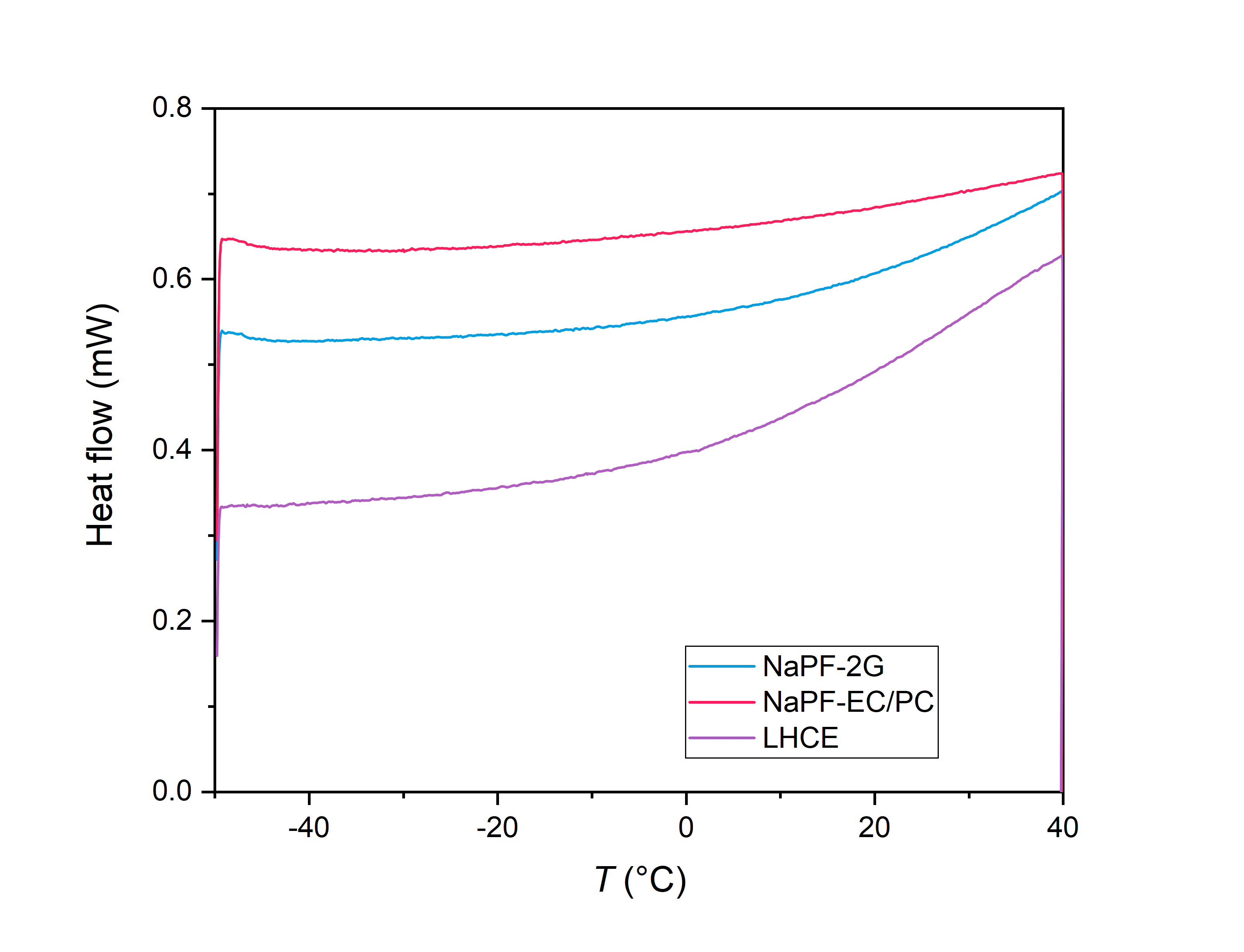


**Figure S2.** DSC results during heating up from -50 °C to 40 °C with a heating speed of 2 °C min^-1^.


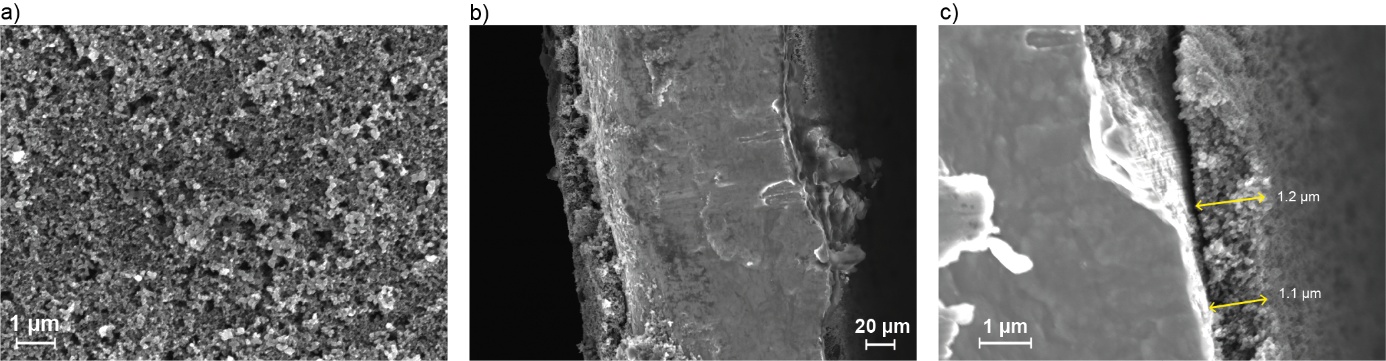


**Figure S3.** SEM images of a) the surface and b-c) cross-section of conductive carbon-coated Al foil. The thickness of the coating is indicated in c).


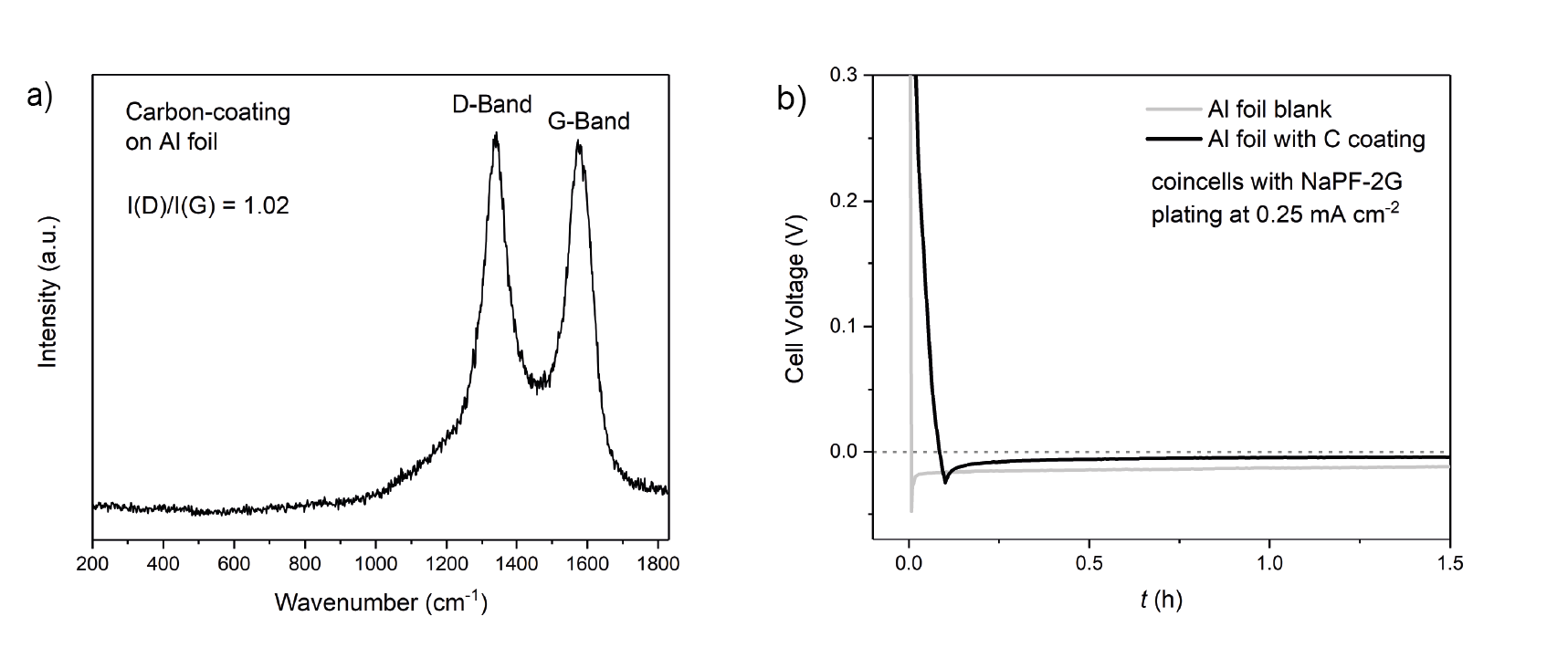


**Figure S4.** a) Raman spectrum of the carbon coating on Al foil, the intensity ratio of the D-band and G-band is 1.02. b) Initial nucleation and plating of sodium on coated (black) and blank Al foil (grey) in coin cells with NaPF-2G and Na as the counter electrode at 0.25 mA cm^-2^.


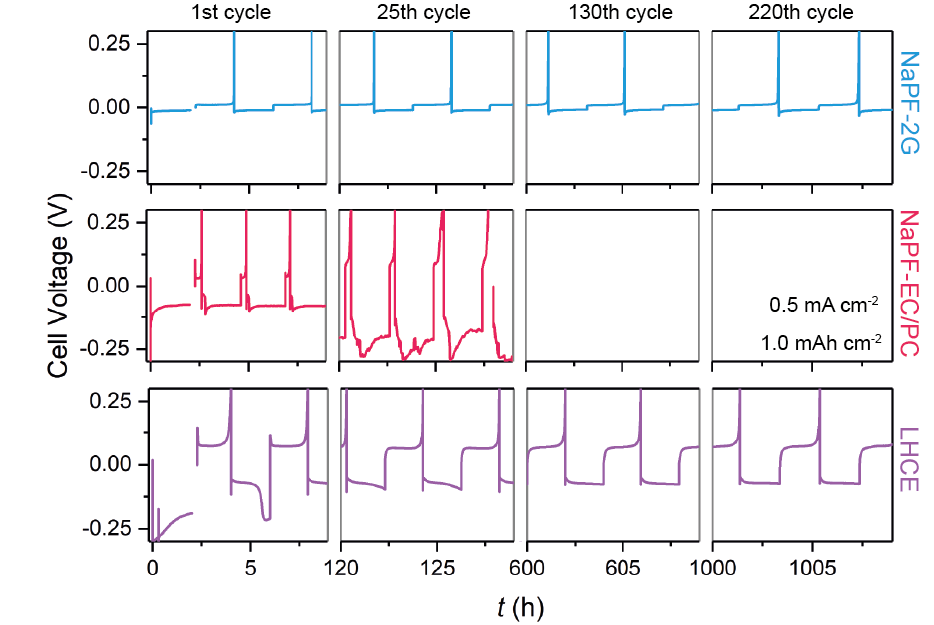


**Figure S5.** Individual potential curves during the long-term cycling of C-Al vs Na in coin cells for the electrolytes NaPF-2G (blue, top), NaPF-EC/PC (red, middle), LHCE (violet, bottom) cycled at 0.5 mA cm^-2^ and 1.0 mAh cm^-2^.


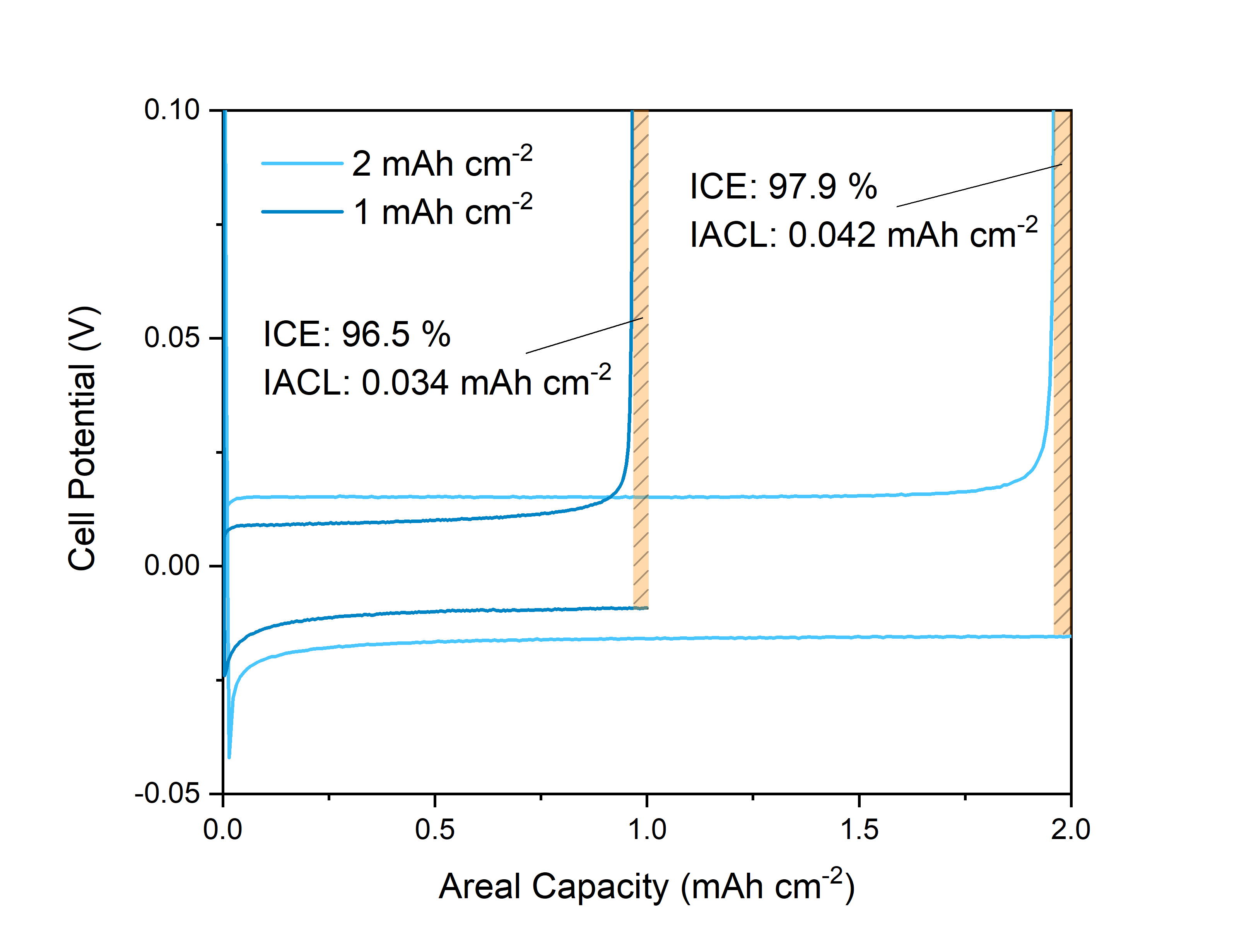


**Figure S6.** Comparison of the areal capacity of the initial cycling of C-Al vs Na in coin cells with the electrolyte NaPF-2G cycles at 0.5 mA cm^-2^ and 1.0 mAh cm^-2^ (dark blue), and at 1.0 mA cm^-2^ and 2.0 mAh cm^-2^ (light blue).


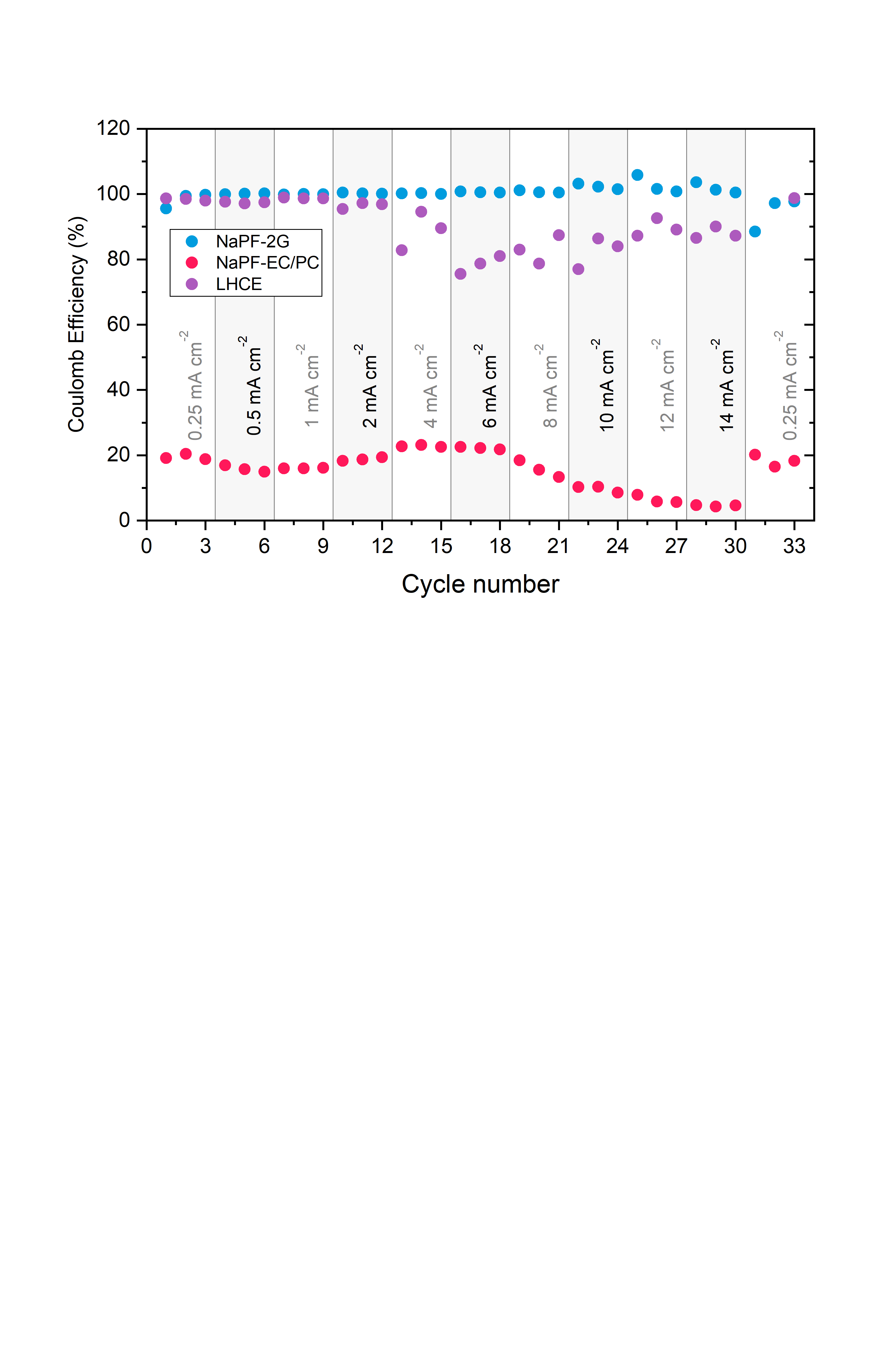


**Figure S7**: Coulomb efficiency of different current densities in C-Al vs Na coin cells for the electrolytes NaPF-2G (blue), NaPF-EC/PC (red), LHCE (violet). At each step, a capacity of 1.0 mAh cm^-2^ is deposited. At high current densities, the cut-off potential of -2.0 V is reached due to increased overpotential before the full 1.0 mAh cm^-2^ could be plated.


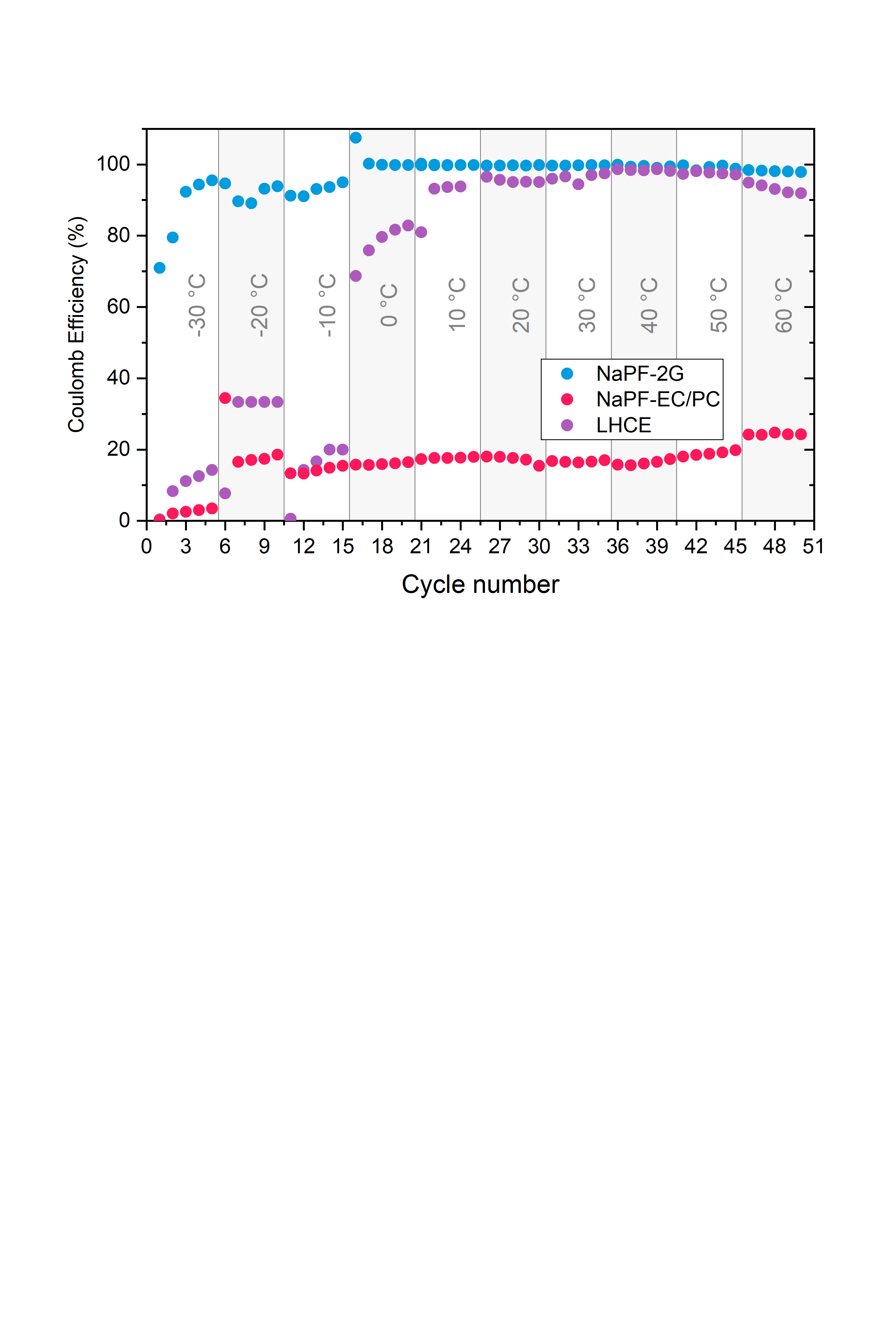


**Figure S8**: Coulomb efficiency of Na plating/stripping at different temperatures in C-Al vs Na coin cells for the electrolytes NaPF-2G (blue), NaPF-EC/PC (red), LHCE (violet). At each step, a capacity of 1.0 mAh cm^-2^ is deposited at a rate of 0.5 mA cm^-2^. At low temperatures, the cut-off potential of -2.0 V is reached due to increased overpotential before the full 1.0 mAh cm^-2^ could be plated.

**
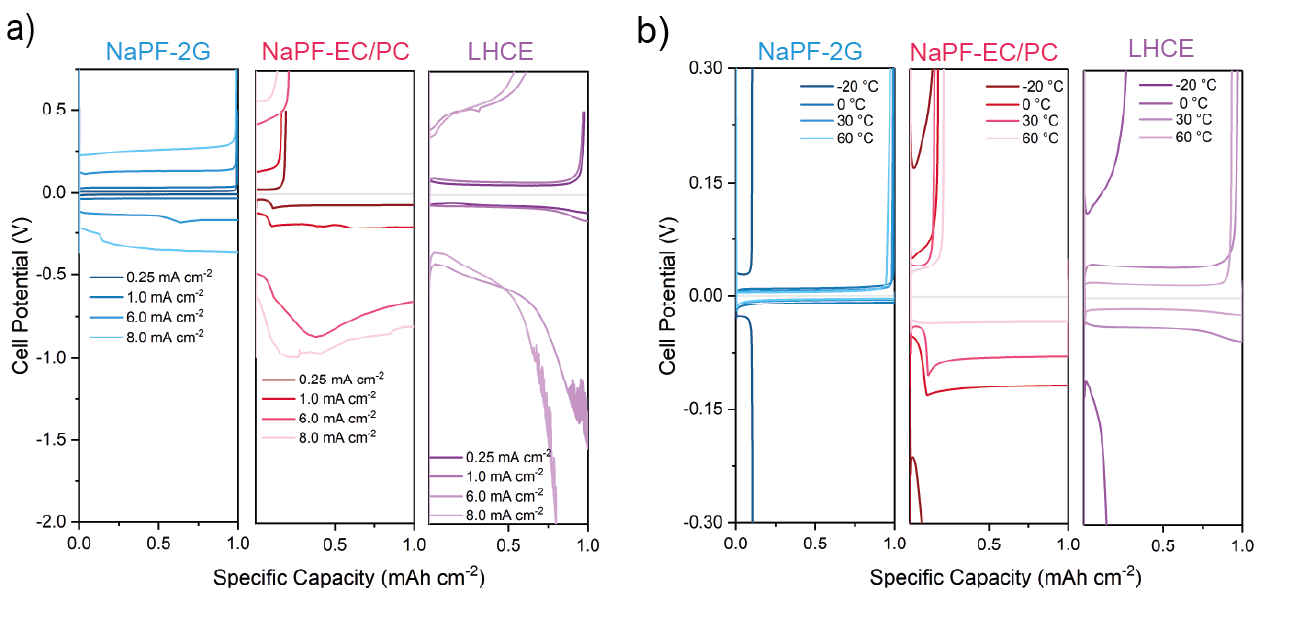
**

**Figure S9**. Potential curves of cycling with a cut off voltage of - 2 V for the electrolytes NaPF-2G (blue, left), NaPF-EC/PC (red, middle), LHCE (violet, right): a) with different current densities from 0.25 mA cm^-2^ till 8 mA cm^-2^ at room temperature and a capacity of 1.0 mAh cm^-2^ and b) at temperature range from - 20 °C to 60 °C cycled at 0.5 mA cm^-2^ and 1.0 mAh cm^-2^.


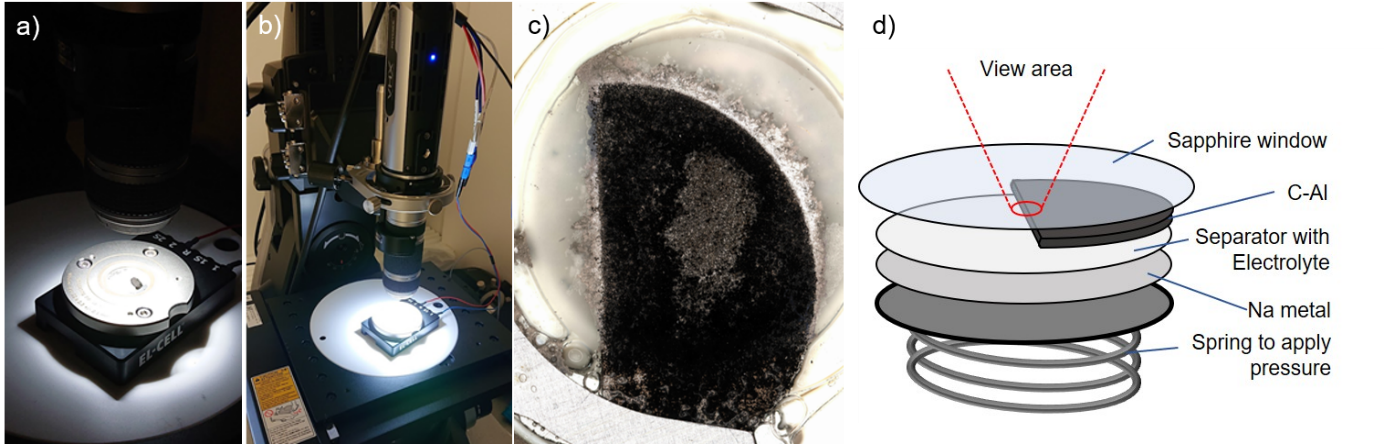


**Figure S10**: The operando optical microscopy setup: a) and b) photographs of the digital optical microscope with the commercial operando cell, c) microscopy picture of the C-Al electrode in plated state at low magnification, d) schematic of the cell arrangement. The C-Al electrode is a circular disk folded in the middle to receive a smooth and coated edge for the plating.


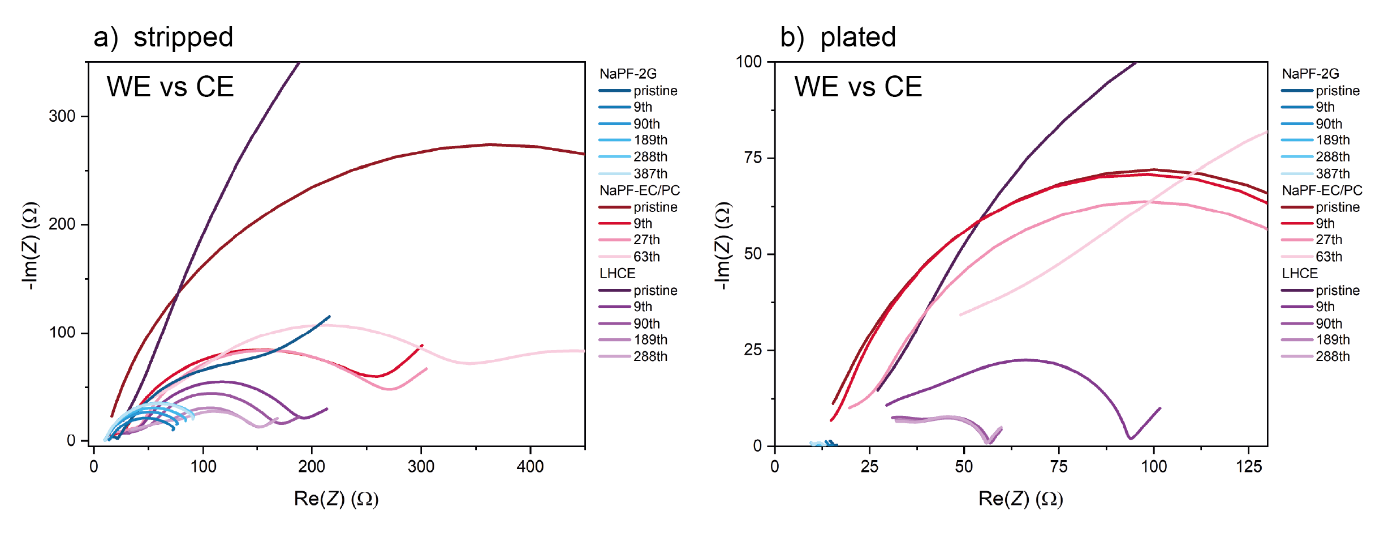


**Figure S11**: Nyquist plot of the impedance in C-Al vs Na coin cells for the electrolytes NaPF-2G (blue), NaPF-EC/PC (red), LHCE (violet, bottom) in a) stripped, and b) plated state cycled at 0.5 mA cm^-2^ and 1.0 mAh cm^-2^.


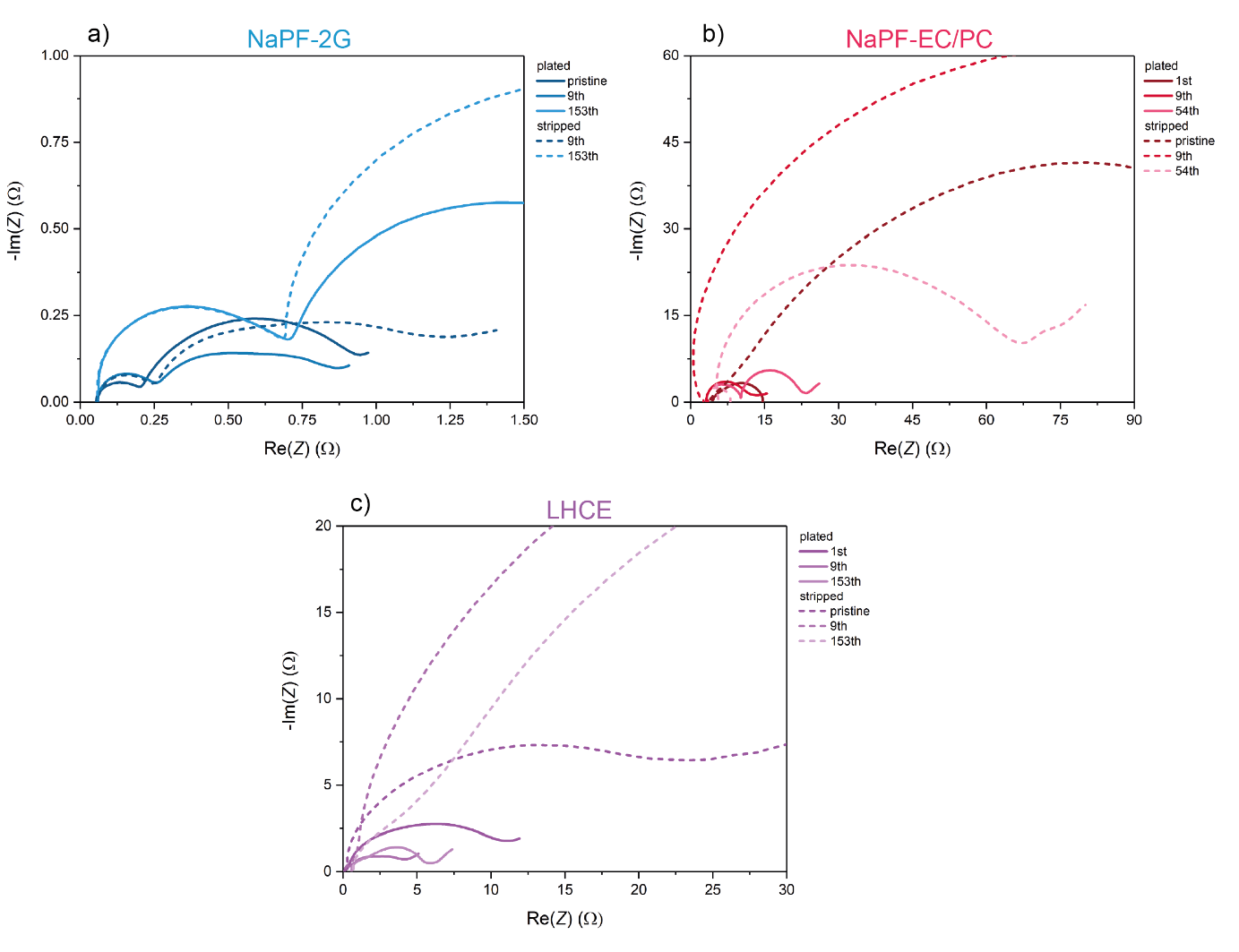


**Figure S12**: Nyquist plots of the impedance of C-Al (WE) vs Na (RE) in three-electrode PAT cells in stripped and plated state with the three different electrolytes, a) NaPF-2G, b) NaPF-EC/PC, and c) LHCE cycled at 0.5 mA cm^-2^ and 1.0 mAh cm^-2^.


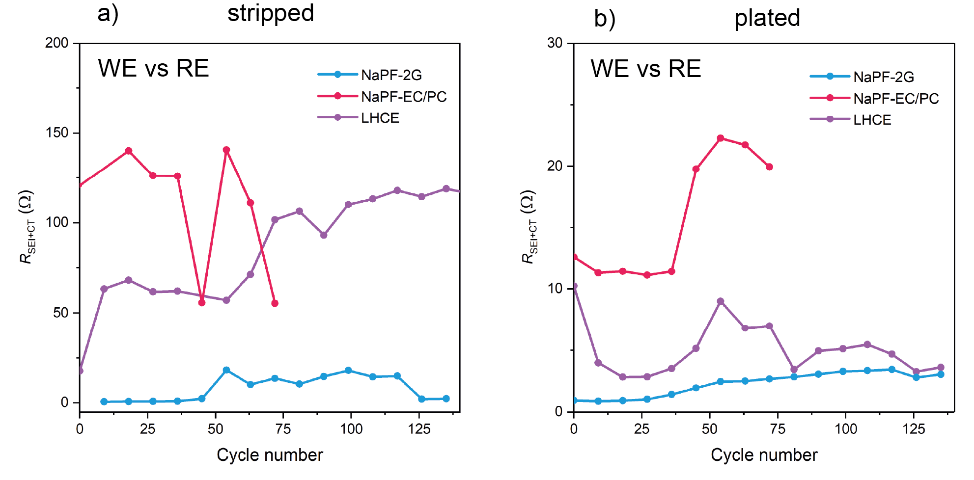


**Figure S13**: Fitted resistance of SEI and CT of C-Al (WE) vs Na (RE) in three-electrode PAT cells in a) stripped, and b) plated state with the three different electrolytes NaPF-2G (blue), NaPF-EC/PC (red), and LHCE (violet) cycled at 0.5 mA cm^-2^ and 1.0 mAh cm^-2^.

**Table S3**: Redox potential, specific capacity, and density of battery components of anode-free cells compared to Na-ion, and Li-ion cells

|  | Redox potential | | Specific capacity | | | | Density |
| --- | --- | --- | --- | --- | --- | --- | --- |
| LFP | 3.4 | V vs. Li^+^/Li | 155 | mAh g^-1^ | 3.47 | g cm^-3^ | |
| NFPP | 3.1 | V vs. Na^+^/Na | 129 | mAh g^-1^ | 3.24 | g cm^-3^ | |
| NFM | 3 | V vs. Na^+^/Na | 170 | mAh g^-1^ | 4.47 | g cm^-3^ | |
| Graphite | 0.2 | V vs. Li^+^/Li | 350 | mAh g^-1^ | 2.25 | g cm^-3^ | |
| Hard Carbon | 0.25 | V vs. Na^+^/Na | 300 | mAh g^-1^ | 1.8 | g cm^-3^ | |
| carbon black |  |  |  |  | 1.5 | g cm^-3^ | |
| binder |  |  |  |  | 1.5 | g cm^-3^ | |
| electrolyte |  |  |  |  | 1.5 | g cm^-3^ | |

**Table S4**: Composition of anode and cathode, porosity, area capacity, and thickness of the current collector and separator.

|  | active material | carbon black | binder | porosity | Area capacity (mAh cm^-2^) | Thickness Al/Cu/Sep (µm) |
| --- | --- | --- | --- | --- | --- | --- |
| cathode | 94% | 3% | 3% | 25% | 3 | 10 |
| anode | 98% | 1% | 1% | 35% | 3.3 | 10 |
| separator |  |  |  | 50% |  | 10 |


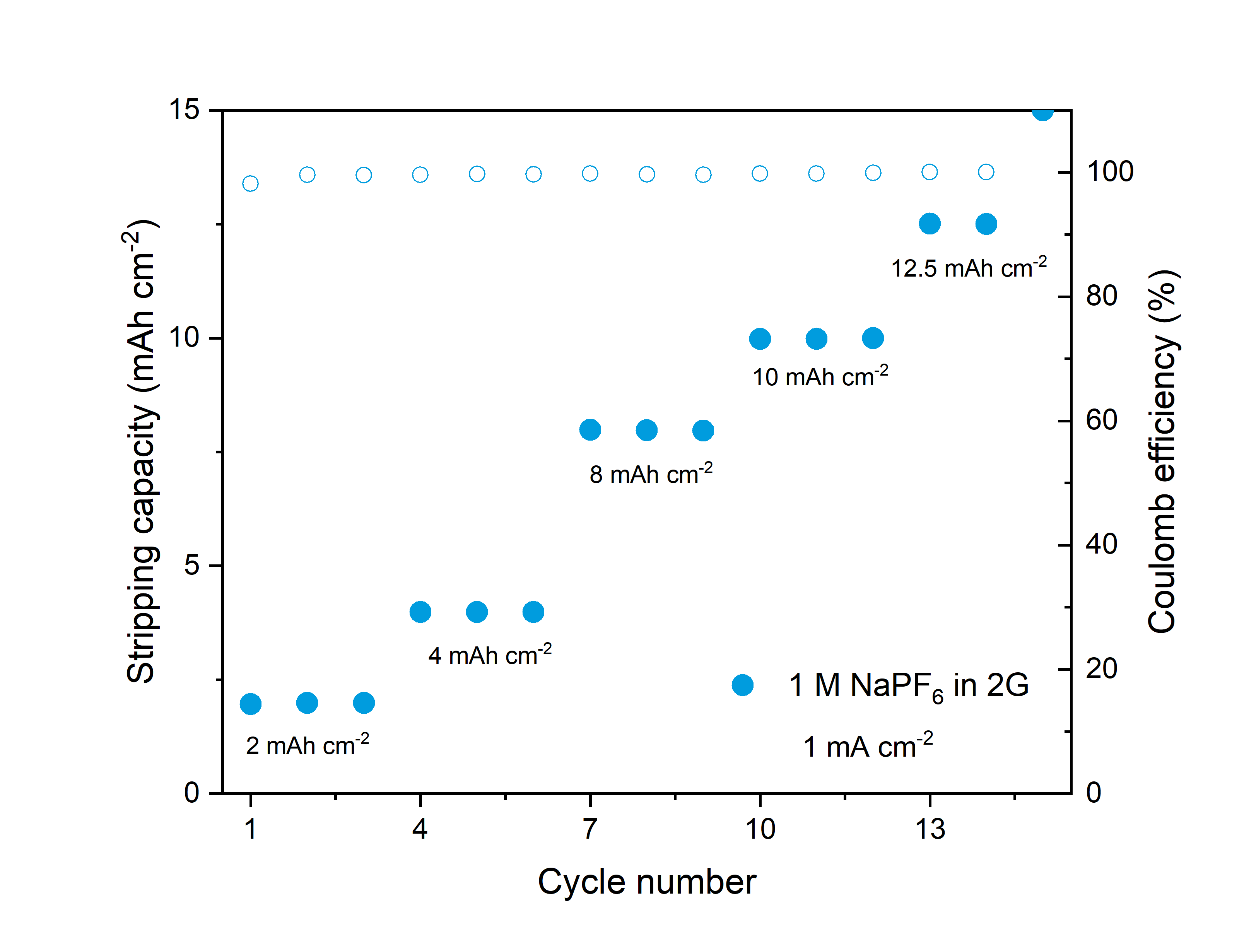


**Figure S14**: Capacity test with NaPF-2G with C-Al (WE) vs Na (AE) in coin cells with 1 mA cm^-2^ and capacities ranging from 2 mAh cm^-2^ to 12.5 mAh cm^-2^. At 12.5 mAh cm^-2^, a short is formed.
